# Comparison of Bacterial and Fungal Composition and Their Chemical Interaction in Free Tropospheric Air and Snow Over an Entire Winter Season at Mount Sonnblick, Austria

**DOI:** 10.3389/fmicb.2020.00980

**Published:** 2020-05-20

**Authors:** Nora Els, Marion Greilinger, Michael Reisecker, Romie Tignat-Perrier, Kathrin Baumann-Stanzer, Anne Kasper-Giebl, Birgit Sattler, Catherine Larose

**Affiliations:** ^1^Institute of Ecology, University of Innsbruck, Innsbruck, Austria; ^2^Institute of Chemical Technologies and Analytics, Vienna University of Technology, Vienna, Austria; ^3^Zentralanstalt für Meteorologie und Geodynamik (ZAMG), Vienna, Austria; ^4^Avalanche Warning Service Tyrol, Department of Civil Protection, Federal State Government of Tyrol, Innsbruck, Austria; ^5^Environmental Microbial Genomics Group, Laboratoire Ampère, École Centrale de Lyon, Écully, France

**Keywords:** Sonnblick Observatory, air-snow comparison, seeding effect, PM10, microbial communities, 16S ribosomal RNA, ITS, 18S

## Abstract

We investigated the interactions of air and snow over one entire winter accumulation period as well as the importance of chemical markers in a pristine free-tropospheric environment to explain variation in a microbiological dataset. To overcome the limitations of short term bioaerosol sampling, we sampled the atmosphere continuously onto quartzfiber air filters using a DIGITEL high volume PM10 sampler. The bacterial and fungal communities, sequenced using Illumina MiSeq, as well as the chemical components of the atmosphere were compared to those of a late season snow profile. Results reveal strong dynamics in the composition of bacterial and fungal communities in air and snow. In fall the two compartments were similar, suggesting a strong interaction between them. The overlap diminished as the season progressed due to an evolution within the snowpack throughout winter and spring. Certain bacterial and fungal genera were only detected in air samples, which implies that a distinct air microbiome might exist. These organisms are likely not incorporated in clouds and thus not precipitated or scavenged in snow. Although snow appears to be seeded by the atmosphere, both air and snow showed differing bacterial and fungal communities and chemical composition. Season and alpha diversity were major drivers for microbial variability in snow and air, and only a few chemical markers were identified as important in explaining microbial diversity. Air microbial community variation was more related to chemical markers than snow microbial composition. For air microbial communities Cl^–^, TC/OC, SO_4_^2–^, Mg^2+^, and Fe/Al, all compounds related to dust or anthropogenic activities, were identified as related to bacterial variability while dust related Ca^2+^ was significant in snow. The only common driver for snow and air was SO_4_^2–^, a tracer for anthropogenic sources. The occurrence of chemical compounds was coupled with boundary layer injections in the free troposphere (FT). Boundary layer injections also caused the observed variations in community composition and chemistry between the two compartments. Long-term monitoring is required for a more valid insight in post-depositional selection in snow.

## Introduction

A major driver of aerobiological research is the assumption that airborne aerosols may act as a seeding source of surface microbes. This is especially presumed for snow and ice covered surfaces in remote areas (Pedgley, 1991; Cowan et al., 2011; Harding et al., 2011; Polymenakou, 2012; Hauptmann et al., 2014), since other sources, such as anthropogenic impacts, are considered minor.

Atmospheric processes of aerosolization, the properties of surrounding landscapes, the lifting of air beyond the boundary-layer to enable long-range transport of microbes from their source regions, the survival of microorganisms in changing temperature regimes of clouds or the ability to survive temperature extremes, as well as desiccation or nutrient limitation, are all factors impacting the composition and dispersion of aerosolized microbes (Möhler et al., 2007; Burrows et al., 2009; Bowers et al., 2011; Xia et al., 2013; Bianco et al., 2016; Carotenuto et al., 2017; Alsved et al., 2018; Evans et al., 2019; Tignat-Perrier et al., 2019). Bertolini et al. (2013) suggested meteorological factors together with the chemical composition of the airborne particulate matter as well as the air mass sources as driving factors for airborne microbial variability at ground level.

It is well known that the origin of the air mass impacts physico-chemical and microbial composition (Amato et al., 2007; Monteil et al., 2014) and that clouds, e.g., air or precipitation including Saharan dust, carry a specific microbial and chemical signature (Chuvochina et al., 2011; Peter et al., 2014; Meola et al., 2015; Weil et al., 2017; Greilinger et al., 2018).

Airborne microbes are known to act as ice nuclei (Christner et al., 2008), essential for precipitation formation, especially the formation of snow. The atmosphere has been suggested as a seeding source for snowpacks (Maccario et al., 2019). During winter deposition and snow cover formation interstitial air containing microbes might be included in the snow cover. The air movement within the snow cover allows an interaction of the entrapped air with the atmosphere over several meters depth (Domine and Shepson, 2002). With more compaction and the formation of ice lamellae within the snow cover, less air movement is possible, thus a direct interaction of the trapped air microbiota with the snow microbiota may occur. Once deposited, post-depositional selection processes occur in the snowpack to form a snow-specific microbial community (Segawa et al., 2005; Maccario et al., 2014).

The snow cover has been considered as a proxy for atmospheric and environmental conditions as well as for bioaerosol microbial composition in previous investigations (Cáliz et al., 2018; Spear et al., 2018; Triadó-Margarit et al., 2019). However, we recently showed that this is not the case for air microbial composition (Els et al., 2019). One of the limitations of aerobiological studies aiming to compare with stationary environmental systems like snow cover is that the air composition changes constantly. Biological air samples are usually only snapshots due to the nature of their limited sampling time. While snow is stationary, though also dynamic and exposed to snow drift and snow blowing (Gallée et al., 2001), it constantly interacts with the atmosphere. Besides the challenge of retrieving representative biological samples of air and snow, the bacterial abundance of one m^3^ air equals one mL of molten snow. Thus, considerable air volumes are needed to reach comparable numbers of bacteria or fungi in snow.

Several studies revealed variability of PM10 aerosol ion composition on a seasonal basis (Perrone et al., 2010; Squizzato et al., 2013). The influence of the season was shown to be more significant than the short term variability of the meteorological parameters on air microbial community within the atmospheric boundary layer (ABL) and the free troposphere (FT) (Brodie et al., 2007; Fierer et al., 2008; Franzetti et al., 2011; Bowers et al., 2012, 2013; Bertolini et al., 2013). The term FT describes air masses well above the planetary boundary layer, where surface friction on the air motion is negligible and laminar air flow dominates. Measurements of atmospheric pollutants within the free-tropospheric air are representative for atmospheric background conditions, in contrast, within the planetary boundary layer, air movement is often turbulent including vertical mixing, thus measurements of atmospheric constituents predominately represent regional or local influences.

In this study we account for the typical weaknesses of air-snow comparisons and compare continuous PM10 aerosol filter samples with snow samples representative of the whole winter accumulation snow cover, all sampled at the remote high alpine site Hoher Sonnblick in the Austrian Alps. We investigated whether air and snow microbial and chemical composition were connected over the winter accumulation period in a pristine free-tropospheric environment. Being aware of seasonality in air microbial and chemical composition, we examined (I) if the air-snow interaction was the same over the winter accumulation period, (II) the significance of the air as a seeding source for bacteria and fungi to the high alpine snow cover, and (III) if the air and snow microbial variability is correlated with chemical composition.

## Materials and Methods

### Study Site

**FIGURE 1:**
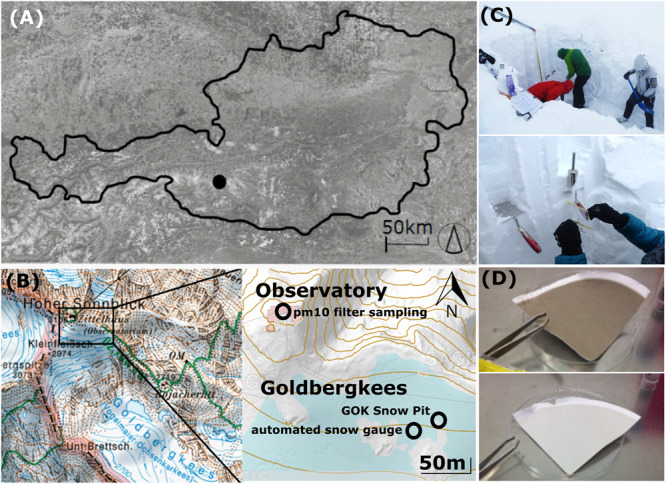
**(A)** Location of the Sonnblick Observatory in Austria, **(B)** location of the PM10 filter sampling (ZAMG Sonnblick Observatory), the snow pit sampling and the automated snow gauge, source: www.salzburg.gv.at/sagisonline, **(C)** snow pit sampling, **(D)** exemplary PM10 quartz air filters for microbial analyses.

Samples were collected at the top of Mount Sonnblick (3106 m asl., Figure 1A), Austria (47°3′14′′ N, 12°57′27′′ E), where a Global Atmosphere Watch meteorological observatory of the World Meteorological Organization (WMO) is operated. The site is exhaust neutral, supplied by electricity from the valley and equipped with an elevated air outlet to enable undisturbed aerosol measurements. The closest settlement is the village Rauris, 15 km away. Rauris village is located at 950 m asl., thus 2156 m below the sampling location on Mount Sonnblick. The area within a 100 km perimeter around the Sonnblick Observatory is sparsely populated.

### Sampling

#### Snow Pit Sampling

A snow pit at the glacier field Goldbergkees (3043 m asl.) on top of Mount Sonnblick was sampled on the 3rd of May 2017 at the end of the winter accumulation period before significant snowmelt began (Figures 1B,C). Snow samples were representative for the whole winter accumulation period and sampling was performed in 10 cm increments (*n* = 31, maximum snow depth of 310 cm). Microbial snow sampling was performed using a stainless steel shovel (ROTH) and sterile nasco whirl-paks (ROTH). A layer of approx. 2 cm above ground was not sampled due to suspected ground contamination and diffusion. Chemical snow sampling was performed in parallel to the microbial sampling within the same pit according to the methods described in Greilinger et al. (2016). Snow stratigraphy and snow density were measured on site (Supplementary Figures S1, S2). Gloves and masks were used to avoid contamination of all samples and the front of the profile was cleared off several times during the sampling. Meltwater percolation or updraft from underlying soil microbes is unlikely since outside temperatures were below 0°C during the snow cover period (Supplementary Figures S3, S4) and the base of the snow profile was solid rock. Microbial and chemical snow samples were kept frozen until their analysis and are named in all further graphs with the snow depth [cm] as increment name.

#### PM10 Quartz Fiber Filter Aerosol Sampling

**FIGURE 2:**
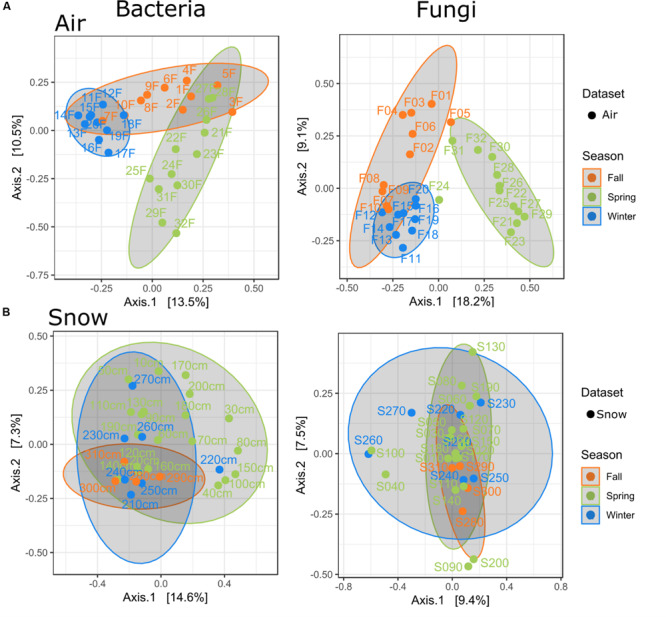
PCoAs for air and snow by season **(A)** only air samples, **(B)** only snow samples, air filters are numbered in order of collection date, while numbers for the snow profile represent depth increments.

Aerosols were sampled on quartz filters (Pallflex Tissuequartz, 2500 QAT-UP, size 150 mm, PALL Life Science Membrane, Figure 1D) using a high-volume sampler (Digitel DHA-80) with a PM10 cut off air inlet according to the European standard reference method EN12341:2014 for PM 10 sampling. The inlet is not heated to avoid losses of semi volatile compounds but single filters which might have been affected by a moisture entry during severe weather conditions were removed. Daily maintenance of the inlet is provided by the staff members continuously present at the site. Additionally, the inlet is surrounded by a wind shield with about 1 m in diameter and about 50 cm height to avoid sampling artifacts during extremely windy and harsh weather conditions, still ensuring a representative inflow into the sampler. Filters were sampled over the course of 1 week with some exceptions (see Table 1). Filters were placed directly from the manufacturers packaging into filter holders which were washed with milli-Q water and stored in a sealed stainless steel cylinder during transport and storage. Once back in the lab, PM10 filters were stored at −20°C until chemical and microbiological analysis. Seasons were allocated to the filter samples according to Greilinger et al. (2016), the snow increments were then allocated to the respective seasons by SNOWPACK modeling (see section “Snowpack Time Allocation Modeling”) and validated with PCoA (Figure 2 and Supplementary Figure S5).

**TABLE 1:** Overview of air filter sampling times and volumes analyzed, if a notable precipitation event took place (indicated by x) or if Sahara event was detected during the sampling period (deduced from the aerosol measurements at the Sonnblick observatory and air mass back trajectories) and the allocated season according to Greilinger et al. (2016).

Filter name	Start [Date, Time]	End [Date, Time]	m^3^ air on Filter	Precipitation	Sahara event	Season
F01	01.09.2016 15:26:18	08.09.2016 12:28:19	1361.29	x	x	Fall
F02	08.09.2016 12:30:40	15:09.2016 12:30:41	1394.24	–		Fall
F03	15.09.2016 12:31:46	22.09.2016 12:31:47	1385.12	x		Fall
F04	22.09.2016 12:40:53	29.09.2016 12:40:57	1383.97	–		Fall
F05	29.09.2016 13:56:14	06.10.2016 13:56:14	1373.93	x		Fall
F06	06.10.2016 14:52:22	13.10.2016 11:52:29	1373.19	x		Fall
F07	20.10.2016 13:12:15	27.10.2016 13:12:15	1368.28	x	x	Fall
F08	27.10.2016 14:32:41	03.11.2016 14:32:41	1369.97	–		Fall
F09	03.11.2016 15:34:30	10.11.2016 12:34:30	1348.28	x		Fall
F10	10.11.2016 12:45:54	01.12.2016 10:04:26	409.14	x		Fall
F11	01.12.2016 10:04:30	08.12.2016 14:56:07	1405.37	–		Winter
F12	08.12.2016 14:56:15	15.12.2017 00:00:00	na	–		Winter
F13	15.12.2017 00:00:00	23.12.2016 16:21:07	2934.85	–		Winter
F14	23.12.2016 16:21:20	30.12.2106 16:21:08	1360.58	–		Winter
F15	30.12.2016 16:21:17		na	–		Winter
F16		12.01.2017 12:26:32	na	x		Winter
F17	12.01.2016 12:26:43	19.01.2017 12:26:31	1359.07	x		Winter
F18	19.01.2017 15:56:37	26.01.2017 12:47:08	1335.70	–	x	Winter
F19	26.01.2017 12:47:16	02.02.2017 13:18:40	1368.25	x		Winter
F20	02.02.2017 13:18:51	16.02.2017 13:44:07	2738.17	x	x	Winter
F21	16.02.2017 13:44:19	23.02.2017 13:25:35	1365.07	x	x	Spring
F22	23.02.2017 13:25:46	02.03.2017 13:01:41	1366.50	x	x	Spring
F23	02.03.2017 13:01:52	09.03.2017 13:11:38	1365.54	x		Spring
F24	09.03.2017 13:11:49	10.03.2017 09:36:00	124.64	x		Spring
F25	10.03.2017 09:36:50	16.03.2017 14:46:22	1203.29	x	x	Spring
F26	16.03.2017 14:46:33	23.03.2017 14:46:21	1369.13	–		Spring
F27	23.03.2017 18:10:27	30.03.2017 13:57:57	1203.29	–	x	Spring
F28	30.03.2017 13:58:10	06.04.2017 13:45:57	1367.94	–		Spring
F29	06.04.2017 13:49:15	13.04.2017 12:00:07	1354.70	–		Spring
F30	13.04.2017 12:00:19	20.04.2017 12:00:07	1339.07	x		Spring
F31	20.04.2017 14:49:47	27.04.2017 14:19:15	1356.74	x		Spring
F32	27.04.2017 14:19:26	04.05.2017 14:19:14	1363.52	x		Spring

### DNA Extraction and Sequencing

Snow was molten at 4°C, filtered through a 0.2 μm polycarbonate filter (47 mm, Isopore) and then stored frozen at −20°C until DNA extraction, performed with the DNeasy Power Water extraction kit (QIAGEN) following the protocol provided with the kit.

From the filters DNA was also extracted using a DNeasy Power Water extraction kit (QIAGEN) using an adapted protocol described in Dommergue et al. (2019) to remove DNA from quartz. Amplification, library prep (MiSeq Illumina sequencing, 2 × 250 bp, Nextera XT Library Preparation Kit) and sequencing was carried out at the Environmental Microbial Genomics group at the Laboratoire Ampère (ECL Lyon, University of Lyon, France). Community diversity was targeted: the V3-V4 region of the bacterial 16S rRNA SSU gene was amplified using 341F/785R primers (S-D-Bact-0341-b-S-17/S-D-Bact-0785-a-A-21, Klindworth et al., 2013) and the fungal internal transcribed spacer (ITS) regions were amplified with primer pair 5.8S_Fung/ITS4 targeting the ITS2 region (Taylor et al., 2016).

### Bioinformatics and Statistics

For 16S sequences, the base quality of the reads 1 and 2 was controlled using the functions fastx_quality_stats and fastq_quality_boxplot_graph of the FASTX-Toolkit^1^. PANDAseq (Masella et al., 2012) was used to assemble read 1 and 2 using the RDP algorithm, the minimum and maximum length of the resulting sequences for 16S were 410 bp and 500 bp, and 390 bp and 500 bp for ITS, with a minimum and maximum overlap length of 20 bp and 100 bp, respectively.

The resulting sequences were stripped of their primers and annotated to the genus or family level by RDP Classifier (Wang et al., 2007) using the RDP 16srRNA and fungallsu databases. Singletons were removed. Negative control OTUs were subtracted for all samples (Supplementary Figure S6 and Supplementary Table S1 for rarefaction curves and sequence statistics). Raw sequences were stored at ftp://ftp-adn.ec-lyon.fr/Els_2020_amplicon_sequencing_air_snow/.

Statistical analyses were done in R (R Core Team, 2015) using the phyloseq (McMurdie and Holmes, 2013), vegan (Oksanen et al., 2018), and ggplot (Wickham, 2009) packages.

Mean raw bacterial and fungal read counts per sample were 20153 (+ −11280) and 20899 (+ −21582), respectively. The whole dataset comprised 1566 bacterial and 3127 fungal genera, which accounted for 1058 bacterial and 1902 fungal genera after removal of blanks.

The statistical analyses were run both rarefied and untreated on the dataset, which did not show differences, but removed rare genera. To include otherwise removed genera and to present the data characteristics appropriately, relative abundance analysis was carried out on non-filtered data (Weiss et al., 2017). Bray-Curtis distances for bacteria and fungi were calculated on datasets normalized to relative abundance (Weiss et al., 2017) and ordinated with principal coordinate analysis (PCoA).

Pairwise PERMANOVA (permutational multivariate analysis of variance) was conducted using the package “pairwiseAdonis” (Martinez Arbizu, 2017), ANOSIM, ADONIS, Chao1, and Shannon indices were calculated using the package “phyloseq”(McMurdie and Holmes, 2013). Bonferroni *p*-value correction (i.e., p.adj) was applied as default for multiple corrections. Statistical parameters of ANOSIM (999 permutations), ADONIS (999 permutations), PERMANOVA and wilcoxon test statistics are reported in the Supplementary Tables S2–S6. The significance level was set to alpha = 0.05.

### Microbial Quantification

To estimate the abundance of bacteria and fungi, both 16S rRNA genes [primer 338F/518R (Øvreås and Torsvik, 1998)] and 18S rRNA genes [primer set FR1/FF390 (Chemidlin Prévost-Bouré et al., 2011)] were quantified by qPCR using Quantifast 2X SYBR Green dye (QIAGEN). Non-template controls were subtracted.

### Chemical Analysis

#### Snow Chemistry

Snow samples were kept frozen until analysis for which they have been carefully molten at 4°C. Conductivity and pH were measured with a conductivity cell and glass electrode. Anions (Cl^–^, NO_3_^–^, and SO_4_^2–^) and cations (Na^+^, K^+^, NH_4_^+^, Ca^2+^, Mg^2+^) were measured by suppressed ion chromatography (Dionex ICS1100 resp. ICS-3000). Methods for the measurements, detection limits and quality control methods are applied according to Greilinger et al. (2016). All obtained chemical values were volumetrically weighted with the snow water equivalent of the respective layer as a function of snow density.

#### PM10 Quartz Fiber Filter Chemistry

Quartz fiber filters were analyzed for different compounds. The carbonaceous fraction of organic, elemental and total carbon (OC, EC, and TC, respectively), were thermal-optically measured. Therefore, a filter punch with 10 mm in diameter was used for analysis with the OC-EC analyzer (Sunset Lab), using the EUSAAR2 reference temperature program and automatic split point setting as performed by the evaluation software.

Anions (Cl^–^, NO_3_^–^, and SO_4_^2–^) and cations (Na^+^, K^+^, NH_4_^+^, Ca^2+^, Mg^2+^) were determined using ion chromatography with the same setup as for the snow samples. Levoglucosan was also measured via ion chromatography (Dionex ICS-3000) but without using a suppressor. For the measurements of the cations a punch of 12 mm in diameter was placed in a polypropylene test tube and 3 mL of 38 mmol MSA (Methanesulfonic acid) were added. The sample was well mixed and placed in an ultrasonic bath (30°C and full power) for 20 min. Afterward the sample was centrifuged at 4000 rpm for 10 min and 1.1–1.5 mL of the eluate was used for analysis. For the measurement of the anions and levoglucosan the same punch size of 12 mm was used and the same extraction method was applied, just the eluent was changed to milli-Q water.

Mineral dust tracer such as Al and Fe were determined using a PANalytical AXIOS advanced wavelength dispersive X-ray fluorescence spectrometer with a rhodium target X-ray tube set at 50 kV with a current of 50 mA, a 20 mm aperture for exposure and an exposure time of 20 s per channel. A detailed methodological description can be found in Greilinger et al. (2019).

#### Canonical Analysis of Principal Components and Stepwise Regression

To evaluate the importance of environmental parameters on the variability of the microbial community a stepwise regression with backward elimination was conducted with “*ordistep*” in vegan (Oksanen et al., 2018) and visualized with a canonical analysis of principal components (CAP) based on Bray-Curtis distance on normalized community data. All biological, chemical and physical environmental parameters for CAP analysis and stepwise regression were z-standardized within the air and snow dataset, respectively to account for different scales of unit. See Table 2 for an overview of all parameters used for the CAP analysis.

**TABLE 2:** Overview over all measured chemical, biological, and physical parameters.

	Air	Snow
Bacterial resp. Fungal abundance	x	x
Shannon	x	x
Chao1	x	x
Season	x	x
NO3	x	x
NH4	x	x
SO4	x	x
Ca	x	x
Cl	x	x
Mg	x	x
Na	x	x
K	x	x
OC	x	
TC	x	
EC	x	
NO2	x	
Levoglucosan	x	
Al	x	
Fe	x	
Precipitation	x	
Snow-Water- Equivalent (SWE)		x
pH		x
Conductivity		x
Density		x

*Analyzed samples for the respective environmental compartment indicated by “x”.*

### Snowpack Time Allocation Modeling

SNOWPACK (Lehning et al., 1999) is a 1D multi-layer snowpack model developed at SLF (Swiss Institute for Snow and Avalanche Research) for avalanche forecasting. It is driven by meteorological data that is filtered through a preprocessor for high alpine weather stations. It can simulate the development of the snowpack during the winter and show modeled snow profiles for a given location and time where meteorological data is available by solving mass and energy exchange equations between the snow cover, atmosphere, vegetation and soil, and treating mass and energy fluxes within these media. It takes into account snow metamorphism, settling, phase changes, wind erosion, water and vapor transport, heat conduction and more. The microscopic parameters of grain size, bond radius, sphericity and dendricity are derived from snow density, temperature and liquid water content for each layer. Based on these results, the macroscopic parameters of thermal conductivity and viscosity that are needed to compute the conservation equations are calculated.

In our simulation, the absolute snow height was used to calculate new snow sums. Neither the snow surface temperature nor the reflected short-wave radiation were measured, so for the upper boundary condition, we used the incoming long wave radiation and parameterized the albedo. This implies working with fluxes rather than temperatures, i.e., Neumann boundary conditions. Radiation data from Mt. Sonnblick used as input was obtained within the ARAD network (Olefs et al., 2016). Snow height was obtained from an automatic TAWES laser snow gauge next to the snow pit sampling site, data validation was carried out using data from daily manual snow height measurements and via quality control algorithms. Wind speed and direction, temperature, pressure and humidity were obtained from the meteorological measurements at the Sonnblick Observatory. Details on calculation and model settings and further references are available at https://gitlab.com/lwd.met/snowpack/sonnblick_2016/wikis/home.

**FIGURE 3:**
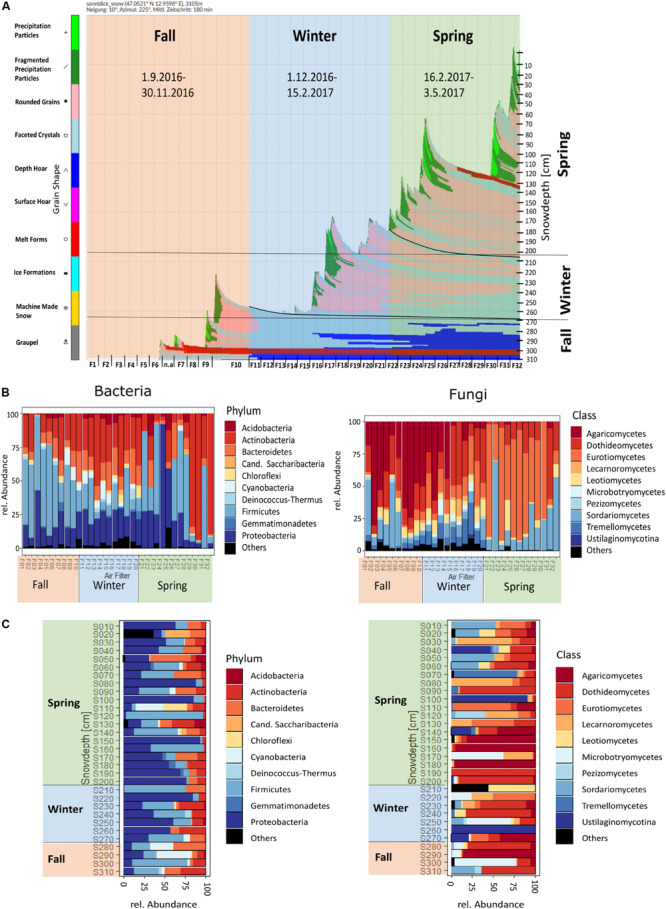
**(A)** SNOWPACK temporal model output and derived seasonal grouping, **(B)** temporal order of air filters, **(C)** profile of snow samples for bacteria and fungi, depicted for the 10 most abundant bacterial phyla and fungal classes.

The results of the simulated profile were compared with the measured grain type, grain size and snow density for the respective layers during field sampling (Supplementary Figures S1, S2). The end-result is the temporal evolution of the snow cover as a time series of modeled snow profiles at our experiment site (Figure 3A).

### Boundary Layer Analyses

Periods with possible transfer of air from the ABL to the Sonnblick Observatory were determined based on time-series of mixing heights. The mixing height is defined as the height above the ground where air and suspended particles from near ground are transported up to by vertical mixing. Exchanges between the ABL and the FT in Alpine areas are caused by advective processes in connection with frontal systems and mountain venting as well as by mixing processes including deep convection, shallow convection, frontal convection and ABL turbulence (Zhang et al., 2018).

The heights of the lowest distinct aerosol layers within the ABL were obtained from backscatter profiles derived from a VAISALA CL51 ceilometer located at Kolm-Saigurn (the base of Mount Sonnblick at 1500 m asl.). Half hourly mixing layer heights were calculated from these aerosol layer heights in combination with wind speed data at 10 m above ground measured at Kolm-Saigurn using the approach described in Lotteraner and Piringer (2016). Cases with mixing heights larger than 1600 m were interpreted as conditions favorable for the transfer of ABL air to or above the top of Mount Sonnblick.

## Results

### Seasonal Dynamics of Bacteria and Fungi in Air and Snow

#### Seasonal Allocation in Air and Snow

The bacterial and fungal communities from air filters and the snow profile differed significantly (ANOSIM bacteria *p* = 0.001, *R* = 0.325, fungi *p* = 0.001, *R* = 0.287). The bacterial community featured 45% of genera unique to the air filters and 45% common in the profile and the air filters, but only 9.8% unique to the snow profile, resp. 82.2% of the snow genera were also present in air. In the fungal community, 74.5% of the genera were unique to the air filters while only 3.4% were unique to the snow profile and 22.1% of genera occurred in air and snow, resp. 86.7% of the snow fungal genera also occurred in the air (see Supplementary Figure S7).

Among the ten most abundant bacterial phyla, the most prominent overall trend was the relative occurrence of more than twice as much Actinobacteria in the air-filters (air 28.09%, snow 10.50%), while Proteobacteria were more abundant in the snow profile (snow 39.15%, air 20.54%). Relative abundances of Bacteroidetes (air 10.11%, snow 12.69%), Chloroflexi (air 1.16%, snow 1.24%), Firmicutes (air 30.29%, snow 26.91%), Gemmatimonadetes (air 0.74%, snow 0.66%) and other less abundant phyla were similar in air and snow. Whereas Cyanobacteria appeared to be twice as common in snow (air 2.56%, snow 4.25%), Acidobacteria were more abundant in air (air 2.64%, snow 1.41%).

For the fungi, Dothideomycetes (air 19.17%, snow 27.16%), Microbotryomycetes (air 1.35%, snow 7.79%), Lecarnomycetes (air 0.93%, snow 10.45%), and Ustilaginomycetes (air 0.32%, snow 9.14%) were more abundant in snow, while Eurotiomycetes (air 27.34%, snow 9.75%) and Sordariomycetes (air 16.49%, snow 7.67%) were more abundant in air. Agaricomycetes (air 20.79%, snow 16.85%) and Leotiomycetes (air 5.97%, snow 4.19%) occurred at comparable relative abundances in air and snow (Supplementary Table S7).

Seasons were determined according to Greilinger et al. (2016) and validated with PCoA clustering of the samples (Figure 2 and Supplementary Figure S5). Snow sample increments were allocated to their respective seasons by SNOWPACK modeling (Figure 3 and Table 3).

**TABLE 3:** Overview of seasonal classification of air filters and snow samples.

	Time	Air Filter	Σ Air Filter	Snow depth	Σ Snow Increments
Fall	1.9-30.11.16	F1–F10	10	310-280	4
Winter	1.12.16-15.2.17	F11–F20	10	270-210	7
Spring	16.2.17-4.5.17	F21–F32	12	200-10	20

Air microbial communities show a seasonal pattern (Figure 2A). Bacterial and fungal winter air samples were the most distinct and closely clustered, with some overlap in winter and fall air samples, but distinctly different to spring samples. The seasonal effect on snow samples was less apparent and showed highest variability in spring for bacteria and winter for fungi and the lowest variability in fall for both (Figure 2B).

In pairwise comparisons (PERMANOVA), the strongest similarity for snow bacteria and fungi was found between spring and winter (p.adj: 1.000), winter and fall (p.adj: 1.000), and fall and spring (p.adj.: bacteria: 0.090; fungi: 0.270). For bacteria, the air and snow autumn communities were the most similar (p.adj.: 0.075), followed by air in spring and the snow in fall and winter (p.adj.: 0.75, resp. 1.000). All other pairwise PERMANOVA comparisons were significantly different (Supplementary Table S3).

The 2016/17 winter accumulation of snow during September to February was 1 m (Figure 3A), during which a 5 week dry period (F10–F15) was observed. During spring, precipitation events led to the accumulation of 2 m of snow, but a 4 week dry period (F25–F29) was also observed, in addition to the formation of an ice lamella. When air filters are presented chronologically, Actinobacteria and Proteobacteria had a peak in spring, while Chloroflexi, Cyanobacteria, Deinococcus–Thermus and Gemmatimonadetes were present only in fall and winter and Firmicutes had a peak in fall. For fungi, Agaricomycetes were high in fall and early winter, while Eurotiomycetes were high in spring. Lecarnoromycetes and Microbotryomycetes occurred only in winter, Tremellomycetes occurred in fall and higher in winter (Figure 3B and see Supplementary Figure S8A for detailed temporal resolution per phyla resp. class). The snow profile showed a more patchy picture of the occurrence of certain phyla and classes of bacteria and fungi (Figure 3C). In winter and spring snow, Proteobacteria were more abundant than in fall, while Cyanobacteria were most abundant in fall snow. The relative abundance of Microbotryomycetes is higher in fall snow and for Leotiomycetes higher in spring snow. In the spring snow Agaricomycetes and some other fungal classes that were not apparent in spring air could be detected (Supplementary Figures S8A–D).

#### Snow Air Comparison by Season and Core Community

**FIGURE 4:**
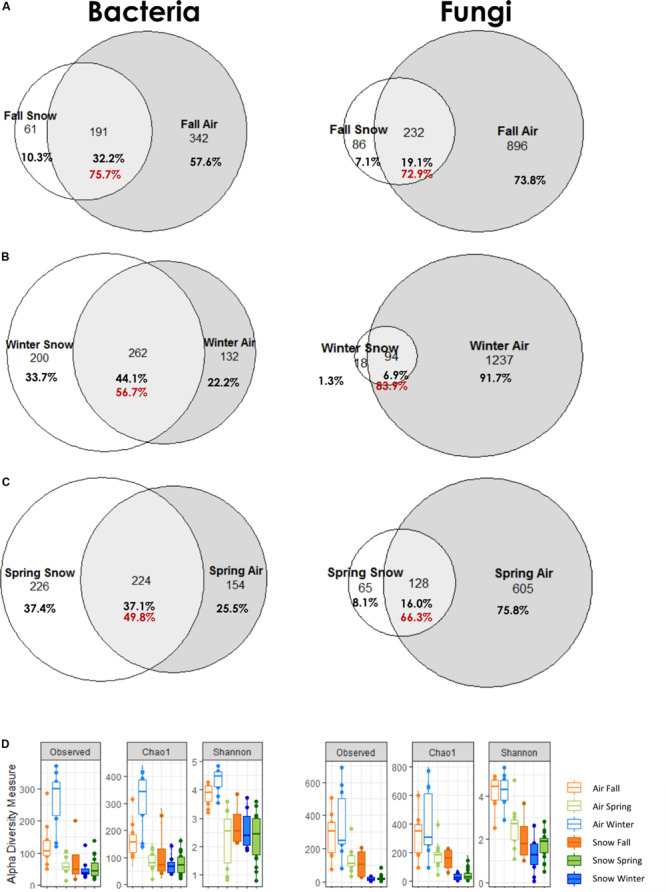
Common genera of air and snow for each season **(A)** fall, **(B)** winter, **(C)** spring; red numbers indicate the percentage of atmosphere genera in the snow dataset; **(D)** alpha diversity measures Observed, Chao1, and Shannon by season for bacteria and fungi.

Comparing bacterial and fungal genera shared between air and snow in the different seasons as a percentage of the snow dataset (Figures 4A–C), the highest overlap was in fall (75.7%) for bacteria, followed by winter (56.7%), and spring (49.8%) and in winter for fungi (83.9%), followed by fall (72.9%), and spring (66.3%). Spring made up the biggest dataset, with the most snow increments (i.e., 20) and air samples (i.e., 12), but had roughly half as many fungal genera in total in air and snow than winter (798 in spring, 1349 in winter, 1214 in fall). Bacteria had roughly the same number of genera in the dataset of each season (594 in fall, 592 in winter and 604 in spring).

Air bacterial diversity was significantly higher in winter (wilcoxon *p* ≤ 0.0002) and no significant differences were observed in bacterial snow alpha diversity across seasons (Figure 4D and Supplementary Tables S4–S6). Fungal alpha diversity was highest in spring and fall air samples for all indicators and highest in fall snow in richness (Chao1, wilcox *p* = 0.018), but did not differ significantly in evenness (Shannon, wilcox *p* = 1.000).

### Chemical Concentrations and Interaction With Microbial Community in Snow and Air

**FIGURE 5:**
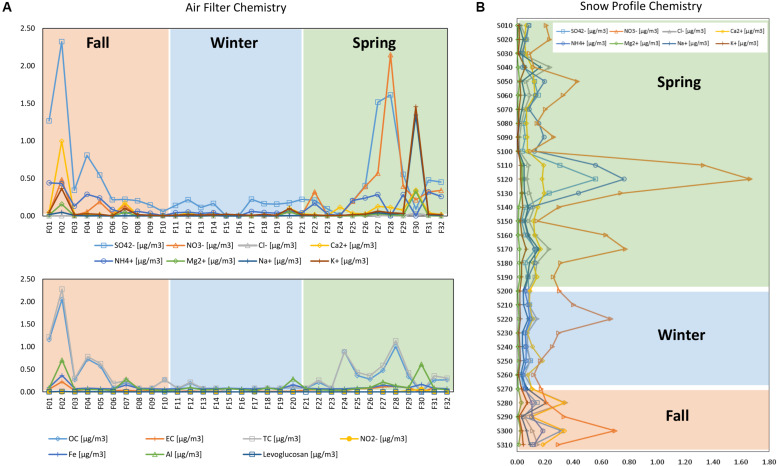
Temporal trends in chemical concentrations in **(A)** air and **(B)** snow profile.

The temporal development of the chemical concentrations showed higher variability in fall and spring in the air filters (Figure 5A), especially for SO_4_^2–^, NO_3_^–^, Ca^2+^, K^+^, and Na^+^. TC and OC were also more variable in the fall and spring (Figure 5A). In the snow profile (Figure 5B), NO_3_^–^, NH_4_^+^, SO_4_^2–^, and Ca^2+^ concentrations were highest in fall and spring, with NO_3_^–^ concentrations varying the most throughout the snow profile.

The backward elimination analysis (Table 4) identified season, alpha-diversity indicators, SO_4_^2–^ and Ca^2+^ as significant factors in explaining variability for the whole dataset. Sample type (i.e., air or snow) and Mg^2+^ were also shown to be significant when included in the analysis. When considering the air samples only, Mg^2+^, Cl^–^, TC/OC, Al/Fe, SO_4_^2–^, season, Chao1 and Shannon index were significant in explaining variability. For the snow only season, Chao1 and SO_4_^2–^ were significant. For fungi the most important factor was season, NO_3_^–^ was significant in the snow dataset, and Shannon diversity was significant in the air.

**TABLE 4:** Importance of environmental parameters on the variability of the microbial community determined by stepwise regression with backward elimination.

Bacteria					Fungi				
	Df	AIC	F	Pr(>F)		Df	AIC	F	Pr(>F)
**Complete snow and air dataset including sampletype as a factor**					**Complete snow and air dataset including sampletype as a factor**				
Ca	1	203.23	1.3309	0.060	Season	3	209.20	2.4461	0.005**
Mg	1	203.27	1.3621	0.060	Type	2	3.9110	3.9110	0.005**
Season	3	202.72	1.3238	0.040*					
SO4	1	203.68	1.7194	0.015*					
Shannon	1	203.81	1.8321	0.005**					
Chao1	1	203.89	1.9021	0.005**					
Type	2	206.52	4.2881	0.005**					
**Complete snow and air dataset not including sampletype**					**Complete snow and air dataset not including sampletype**				
Ca	1	205.54	1.3660	0.045*	Season	3	211.08	2.4034	0.005**
SO4	1	205.84	1.6450	0.020*					
Shannon	1	205.96	1.7517	0.010**					
Season	3	205.56	1.6155	0.005**					
Chao1	1	206.23	2.0023	0.005**					
**Air dataset**					**Air dataset**				
Mg	1	77.683	1.5469	0.015*	Shannon	1	77.292	1.7145	0.030*
Cl	1	77.772	1.6122	0.015*	Season	3	79.856	3.1350	0.005**
TC/OC	1	77.963	1.7540	0.015*					
SO4	1	78.421	2.0960	0.010*					
Season	3	77.773	1.5680	0.005**					
Chao1	1	77.996	1.7786	0.005**					
Fe/Al	1	78.006	1.7860	0.005**					
Shannon	1	78.334	2.0310	0.005**					
**Snow dataset**					**Snow dataset**				
Chao1	1	79.635	1.6228	0.040*	Season	3	83.486	1.2074	0.075.
SO4	1	79.395	1.4096	0.010**	NO_3_	1	84.290	1.3024	0.030*
Season	3	79.603	1.7163	0.005**					

Statistical significance level: *p ≤ 0.05, **p ≤ 0.01.

**FIGURE 6:**
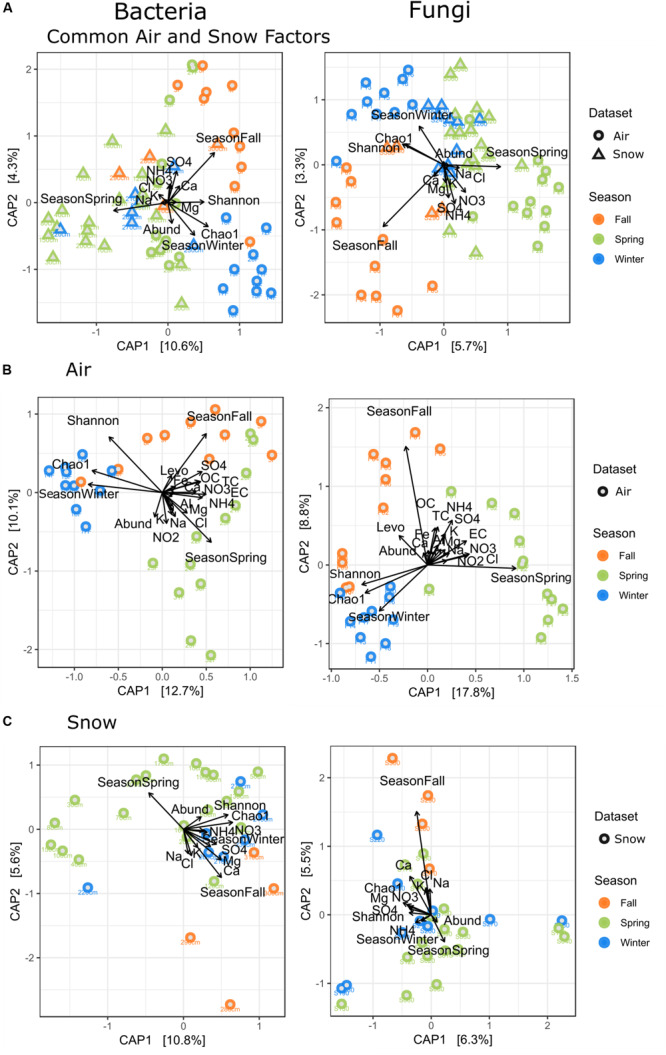
Canonical analysis of principal components (CAP) of all factors measured for **(A)** all factors measured for air and snow, i.e., Season, NO_3_, Cl, NH_4_, SO_4_, Ca, Mg, Na, K, Chao1, Shannon, Abundance, Sampletype, **(B)** for all factors measured for air, i.e., Seasons, NO_3_, Cl, NH_4_, SO_4_, Ca, Mg, Na, K, Chao1, Shannon, Abundance, OC, TC, EC, NO_2_, Levoglucosan, Al, Fe, Precipitation, **(C)** for all factors measured for snow, i.e., Season, NO_3_, Cl, NH_4_, Ca, Mg, Na, K Chao1, Shannon, Abundance.

Canonical analysis of principal components analysis for the whole dataset (Figure 6A) shows that SO_4_^2–^ was related to spring and fall air, while the Chao1-diversity was mostly linked to winter air samples and Shannon-diversity to winter and fall air samples. The whole dataset had 11.5% variability explained in the first two axes, but 14.9% when sample type was taken into account. OC/TC are related to fall and some spring filters, while Mg^2+^ and Cl^–^ are related to spring filters and Al/Fe to spring and fall. Air filters had the highest variability explained by the measured variables (i.e., 22.8% resp. 26.6% for the first two axes, Figure 6B).

The snow (Figure 6C) showed a slight grouping by season for the fall and winter samples, with only the S220 sample grouping with spring samples. Spring samples were most scattered and partially overlapped with winter snow samples. SO_4_^2–^ in snow correlated with fall and winter samples, Chao1 correlated with winter and some spring samples.

SWE, density, conductivity and pH are not displayed, as they had the highest *p*-values (most insignificant *p* values) for snow. Snow had 15.2% resp. 11.8% explained variability for the first two axes.

### Influence of Exchange Between Atmospheric Boundary Layer and Free Troposphere

**FIGURE 7:**
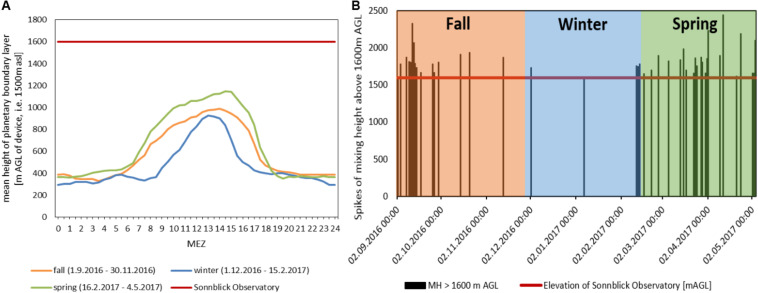
**(A)** Mean height of planetary boundary layer above ground of measuring device (Kolm-Saigurn 1500 m asl), Sonnblick Observatory at 1600 m AGL, **(B)** half hour values of mixing heights [m AGL] reaching the Sonnblick Observatory.

Figure 7A illustrates the mean daily courses of the mixing height for the seasonal periods of investigation. Mixing heights on average were lowest during the winter period (up to 900 m AGL on average, i.e., 2400 m asl.). Due to seasonal position of the sun and the shading by the mountains, the mixing height started to rise up to 2 h later in the morning in winter-time and decreased with sunset up to 2 h earlier than in spring and fall. In spring, the mean mixing height during daytime rose to 1150 m AGL (i.e., 2650 m asl). The observatory is located at 1600 m AGL (i.e., 3100 m asl.), thus the average mixing heights during the investigated periods remained well below the sampling site.

All events with half hourly mixing heights of more than 1600 m are indicated by black vertical lines in Figure 7B, revealing when air might have been transported from the ABL up to the FT. These events occurred most frequently in early fall and in spring. The highest number of mixing layer half hour events reaching the observatory was recorded for fall Filter F02 followed by spring filters F27 and F28 (Table 5). These filters also showed the most prominent peaks in chemical concentrations and sum of ions (Figures 5A,B). The strongest correlation for both mixing height half hour sums between concentration values and the number of half hourly mixing heights above 1600 m was observed for EC > SO_4_^2–^ > Fe > TC > Ca^2+^ > OC, linking the influence of ABL air transported upward to the concentration of chemicals measured at the mountain station.

**TABLE 5:** Sum of mixing heights reaching the Sonnblick Observatory, pearson correlation of sum with concentration of chemical species on the PM10 quartz air filters.

Season	Filter	Number of half hour spikes of mixing height above the observatory	Pearson correlation *r*^2^	PM10 chemical conc. ~Σhalf hour spikes
**Fall**	F01	8	**SO_4_**	**0.7331**
	F02	65	**NO_3_**	0.1811
	F03	11	**Cl**	0.0015
	F04	15	**Ca**	**0.5652**
	F05	13	**NH_4_**	0.3858
	Fxx	6	**Mg**	0.0729
	F06	5	**Na**	0.0073
	F10	4	**K**	0.0081
**Winter**	F11	4	**OC**	**0.5573**
	F15	1	**EC**	**0.7889**
	F20	17	**TC**	**0.6019**
**Spring**	F21	7	**NO_2_**	0.0086
	F22	3	**Fe**	**0.6448**
	F23	9	**Al**	0.3494
	F25	8	**Levoglucosan**	0.4624
	F26	13		
	F27	34		
	F28	21		
	F29	19		
	F31	10		
	F32	9		

*In bold: values >0.5.*

## Discussion

### Is Air a Bacterial and Fungal Seeding Source to Snowpacks?

The atmosphere has been suggested as a seeding source for snowpacks given that snowflakes form in the atmosphere and that the two ecosystems are connected (Maccario et al., 2019). Based on the venn diagram analysis, as the snowpack established in the fall, it shared almost 76% of the identified bacterial and 72.9% of fungal genera with the atmosphere (Figure 4), suggesting a strong interaction between the two compartments. As the snowpack evolved throughout the winter and spring, the overlap with the atmosphere diminished for bacteria (winter 56.7% and spring 49.8%), but increased during the winter for fungi (83.9%) and was lowest for fungi in spring (66.3%). Based on shared genera data, it appears that the snow is seeded by the atmosphere, however, when looking at community structure, the PCoA and statistics reveal a separation of air and snow bacterial and fungal genera. These differences suggest that a distinct air microbiome might exist that is not incorporated in clouds and thus not precipitated or scavenged in snow, or that post-depositional selection of low abundance airborne microorganisms occurs over time within the snowpack, as has previously been suggested (Segawa et al., 2005; Larose et al., 2010; Hell et al., 2013; Maccario et al., 2014). For example, Cyanobacteria were twice as common in snow than in air and have been suggested as being especially successful in establishing in the snow (Harding et al., 2011; Maccario et al., 2019).

In the few available datasets comparing air and snow microbial communities (Šantl-Temkiv et al., 2018; Els et al., 2019; Triadó-Margarit et al., 2019), a comparable or higher diversity in snow samples was generally observed. However, our results indicate a higher richness and evenness of bacterial and fungal organisms in the air, even though the capturing efficiency of the DIGITEL DHA80 (PM10High Volume sampler) for the size of free floating bacteria (approx. 0.2 μm) is likely not 100% due to losses at the inlets. We also expect losses from the removal of DNA from the quartzfiber-filter, due to the DNA binding characteristic of the quartz (Dommergue et al., 2019). Considering the expected losses of bacterial diversity by sampler size cut-off and DNA-extraction losses, the results of higher alpha diversity values in the air samples point toward an even higher expected air alpha diversity. It is possible that the sampling time of 1 week integrated different atmospheric weather processes, thus maximizing the potential for sampling diversity.

### Do Deposition Pathways and Changes in Meteorology Affect Snow Community Structure?

Among all the seasons sampled, spring snow was the most diverse and also had the highest amount of unique bacterial genera. This might be related to the snow fall event that made up samples S010-S130 of the total snow profile that fell in the last 3 weeks before sampling (F30–F32). Microorganisms in precipitation events are scavenged from the air (Franz and Eisenreich, 1998; Maria and Russell, 2005; Zhang et al., 2013), which might explain the higher diversity and variability observed in the spring samples. Snowflakes form in the atmosphere around particles, among them also ice nucleation active bacteria (Christner et al., 2008) and are then deposited. During precipitation, interstitial air, also containing microbes, is included in the snowpack, and can interact with the atmosphere over several meters depth (Domine and Shepson, 2002). During precipitation, snow crystals and supercooled liquid droplets can scavenge gas phase and aerosol species from the atmosphere, which are then introduced to the snowpack (Paramonov et al., 2011; Budhavant, 2014; Wang et al., 2014). In the snowpack, complex and not fully understood chemical conversions, enrichments and release of a range of chemical compounds take place. Snow is especially reactive to UV-radiation and photo-oxidation (Grannas et al., 2007; Domine et al., 2008). These factors may render the snowpack selective for certain types of microorganisms.

As shown in Els et al. (2019), there is a significant difference in the genera deposited with precipitation and present in air, which might also explain the higher amount of unique genera in the snow relative to the atmosphere. The unique snow genera might also be explained by DNA extraction and sequencing depth (Klindworth et al., 2013; Mizrahi-Man et al., 2013; Kennedy et al., 2014) or different seeding sources that were not sampled here.

In the winter, a long dry period was observed (F11–F15) and air masses were stable in terms of boundary layer dynamics. Winter air microbial communities were likely mainly characterized by long range transport, as little exchange or emission and boundary layer injections occurred. Winter air was most separate in bacterial diversity from snow and other groups (Figure 2A) and overlapped only with some fall samples (F07, F10). During the sampling of F07, a Sahara dust event occurred (see Table 1). These depositions are associated with long range transport of air and particles from northern Africa. Given the lack of precipitation events, dry deposition of microorganisms was probably the dominant colonization pathway to the snowpack. Fungi probably settle better in dry deposition, as they are bigger and have a higher density than bacteria (Smith et al., 2011). Their dispersal in the air is closely linked to the aerodynamic diameter of their spores (Yamamoto et al., 2012; Woo et al., 2018), but the most important factor is the seasonal availability of their spores due to their lifestyle (Pickersgill et al., 2017). The results from our study support this hypothesis with fungal communities exhibiting high dynamics in the fall that is not observed in winter or spring when the ground is snow-covered and higher boundary layer dynamics occur.

### Are Microbes and Chemical Compounds Co-transported in Air Masses, and Thus Exhibit the Same Co-correlations in Air and Snow?

To connect air chemistry with air microbiology is challenging. In the case of the presented dataset, the chemistry was analyzed directly from the same filter as the microbiological analyses. The advantage of this is that all differences in sampling procedure, losses in pipes and inlets, cut-off sizes and the general filter sample handling and potential introduced biases are identical for the microbial and chemical sampling until the laboratory stage. The limitation of the method is that the filter samples represent a time integration over the duration of 1 week. This means that short peaks of chemical or microbial atmospheric events would likely not be detected in the samples, thus the data represents only major trends (Tignat-Perrier et al., 2019).

This can also be seen as an advantage in aerobiological sampling, as a range of studies stated high short-term variability of air microbial composition with little observable patterns (Fierer et al., 2008; Polymenakou and Mandalakis, 2013).

Our data shows that season and alpha diversity were the major drivers in microbial variability in snow and air, if regarded together or separately for each sample type, and only a few chemical markers could be identified as important in explaining the microbial variability. The variability explained by chemistry and season was generally low, the highest values were found for the air dataset alone (23.2% air bacteria, 26.8% for fungi), snow and air together reached the lowest explained values, indicating that different factors were driving microbiology in air and snow. Seasonal variability in airborne communities has been recurrently shown within the ABL and in the FT (Brodie et al., 2007; Fierer et al., 2008; Franzetti et al., 2011; Bowers et al., 2012, 2013; Bertolini et al., 2013). As a higher share of variability could be explained by the measured parameters in air as compared to snow, this suggests that different processes are important for the microbial composition and variability in snow than the sampled parameters.

The strongest correlation between the chemical concentrations on the PM10 quartzfiber air filter and the mixing height half hour sums of elevation above the observatory was observed for EC > SO_4_^2^ > Fe > TC > Ca^2+^ > OC implying that ABL air transfer to the mountain tops and to the FT is of high importance for the concentration and composition of inorganic aerosols in the free tropospheric atmosphere. Mountainous terrain exerts a profound influence on aerosol distribution in the atmosphere, with often enhanced mixing and transport processes compared to horizontally homogeneous terrain. Mechanisms like entrainment, advective venting, mountain venting or mountain cloud venting facilitate exchange of ABL air and the FT (De Wekker and Kossmann, 2015). The transport of gases and particles into a tropospheric cloud layer (Cotton et al., 1995) by mountain cloud venting is a particularly effective of these processes (Kalthoff et al., 2013). In air Cl^–^, TC/OC, and SO_4_^2–^ as well as Mg^2+^, and Fe/Al and were identified as related to bacterial variability. At the investigated site the former components are related to anthropogenic activities whereas the latter ones are related to dust particles according to Pio et al. (2007) and Greilinger et al. (2016), implying that dust event related variation of microbes or human impact (SO_4_^2–^, TC/OC, Fe) are driving variability of the air microbial community in the FT.

Ca^2+^, Mg^2+^, and SO_4_^2–^ were also identified driving the bacterial variability in the snow and air dataset. Ca^2+^ and Mg^2+^ are related to the occurrence of bigger crustal parts that might be easier deposited and thus important for air and snow microbial composition and are especially affected by Saharan dust inputs (Greilinger et al., 2018). Bacterial abundance is positively correlated to Ca^2+^ in snow (Margesin and Miteva, 2011). Atmospheric bacterial supply to the snowpack and mineral particle concentration in the snowpack are positively correlated (Segawa et al., 2005). SO_4_^2–^ and Ca^2+^ in aerosols can have multiple sources (Li et al., 2006).

SO_4_^2–^ was the only chemical factor being identified as significant to explain bacterial variability in air and snow and when both regarded together. Sulfate in the atmosphere originates from anthropogenic sources like fuel burning or smelting (Forbes and Garland, 2016). SO_4_^2–^ can also originate from sea salt or biogenic emission of dimethylsulfide and other reduced gases from the ocean, but also volcanic eruptions (Harder et al., 2000), but these are unlikely sources for the atmosphere sampled in this study. The tropospheric residence time of SO_4_^2–^ is 12 days (Harder et al., 2000). Gandolfi et al. (2015) found a significant correlation of SO_4_^2–^, Mg^2+^ and Ba with the microbial community structure of bioaerosols, but could not assess whether this was due to co-variation or taxa specific selection (Baumgartner et al., 2006; Dillon et al., 2007). SO_4_^2–^ is a significant driver of high alpine soil bacterial community composition, particularly in fall (Lazzaro et al., 2015).

Fungi in air were only related to seasonal factors. NO_3_^–^ was the only chemical parameter identified as important for fungal composition in snow. The NO_3_^–^ radical is highly reactive with inorganic and organic compounds in cloud water (Herrmann et al., 1994). Thus, surface reactions of NO_3_^–^ with certain fungal species in the cloud could be a possible explanation for this interaction, as fungal spores were recently identified as major input source of sodium salt in the amazon due to hygroscopic interactions at their surface (China et al., 2018). Fungi can oxidize NH_4_^+^ to NO_3_^–^ in grassland soils (Laughlin et al., 2008), thus also a co-emission of NO_3_^–^ and fungal spores from the same sources within the ABL might be possible. Fungi in alpine soil are more determined by environmental factors (i.e., soil dry weight, altitude, plant species, soil temperature) than chemical composition (Lazzaro et al., 2015).

## Conclusion

In this study we compared the bacterial and fungal communities in continuous PM10 air samples over the entire winter season with a snow profile of the entire winter season in terms of microbial and chemical patterns in the FT.

Most genera found in snow occur also in air, this is more pronounced for fungi than for bacteria (49.8–75.7% for bacteria, 66.3–83.9% for fungi), implying that snow is more similar to air, than air is to snow, thus air microbial composition likely effects snow community, but evolves differently over the seasons.

Fungi share less genera in air and snow than bacteria and generally feature a high unique share of air genera in total and seasonal air. The shared air-snow communities are most different for bacteria in spring and fungi in fall, implying different dispersal patterns. Fungi show a strong seasonal transition of abundant classes in air that is not depicted in snow.

The measured chemical tracers were more important for bacterial variability in air than in snow, either because chemicals in the snowpack are not important for post-depositional microbial selection or not the right compounds were measured. The differentiation between air and snow samples and seasonal (i.e., long-term) impact, and with it, changing alpha-diversity, are more important than chemical markers to explain variation in the bacterial and fungal microbial community in air and snow.

SO_4_^2–^ was identified as an important chemical compound for bacterial composition in air and snow (next to Ca^2+^ and Mg^2+^ dust markers). Fungal composition is not explained by chemical variation, apart from NO_3_^–^ in snow.

Peaks in transported chemicals in the air correlated with increased mixing layer heights rising above mountain top level. Not only inorganic, but also biological aerosols are transferred to the FT with ABL air mixed into the FT at the upper boundary of the convective mixing layer. This mechanism of convective mixing of boundary layer air into the FT seems to be an important factor for microbiological particles going into long-range transport.

The strong seasonal variation and transition of bacterial and fungal composition in air and snow highlight the need for long-term monitoring of bioaerosols to gain insights on their occurrence and reveal how little reliability can be drawn from ABL short-term sample campaigns.

## Data Availability Statement

Raw sequences analyzed in this study were stored under the link: ftp://ftp-adn.ec-lyon.fr/Els_2020_amplicon_sequencing_air_snow/. Details on SNOWPACK calculation and model settings and further references are available at: https://gitlab.com/lwd.met/snowpack/sonnblick_2016/wikis/home.

## Author Contributions

NE conceived the setup, did snow sample fieldwork, conducted the microbial lab work, analyzed the data, and wrote the manuscript. MG and AK-G did the snow profile sampling for chemical analyses, provided the quartz air filters, chemical analyses, a range of feedback on the analyses and the manuscript. MR did the SNOWPACK modeling. RT-P provided guidance with the microbial lab work and ran bioinformatical analyses. KB-S provided boundary layer data. AK-G and BS provided funding. CL supervised the microbial labwork and data analysis and contributed highly to the manuscript.

## Conflict of Interest

The authors declare that the research was conducted in the absence of any commercial or financial relationships that could be construed as a potential conflict of interest.

## Footnotes

1 http://hannonlab.cshl.edu/fastx_toolkit/

## References

[B1] Alsved M. Holm S. Christiansen S. Smidt M. Ling M. Boesen T. (2018). Effect of aerosolization and drying on the viability of *Pseudomonas syringae* cells. *Front. Microbiol.* 9:3086. 10.3389/fmicb.2018.03086 30619167 PMC6305290

[B2] Amato P. Parazols M. Sancelme M. Mailhot G. Laj P. Delort A.-M. (2007). An important oceanic source of micro-organisms for cloud water at the Puy de Dôme (France). *Atmos. Environ.* 41 8253–8263. 10.1016/j.atmosenv.2007.06.022

[B3] Baumgartner L. K. Reid R. P. Dupraz C. Decho A. W. Buckley D. H. Spear J. R. (2006). Sulfate reducing bacteria in microbial mats: changing paradigms, new discoveries. *Sediment. Geol.* 185 131–145. 10.1016/j.sedgeo.2005.12.008

[B4] Bertolini V. Gandolfi I. Ambrosini R. Bestetti G. Innocente E. Rampazzo G. (2013). Temporal variability and effect of environmental variables on airborne bacterial communities in an urban area of Northern Italy. *Appl. Microbiol. Biotechnol.* 97 6561–6570. 10.1007/s00253-012-4450-4450 23053100

[B5] Bianco A. Voyard G. Deguillaume L. Mailhot G. Brigante M. (2016). Improving the characterization of dissolved organic carbon in cloud water: amino acids and their impact on the oxidant capacity. *Sci. Rep.* 6:37420. 10.1038/srep37420 27876758 PMC5120292

[B6] Bowers R. M. Clements N. Emerson J. B. Wiedinmyer C. Hannigan M. P. Fierer N. (2013). Seasonal variability in bacterial and fungal diversity of the near-surface atmosphere. *Environ. Sci. Technol.* 47 12097–12106. 10.1021/es402970s 24083487

[B7] Bowers R. M. McCubbin I. B. Hallar A. G. Fierer N. (2012). Seasonal variability in airborne bacterial communities at a high-elevation site. *Atmos. Environ.* 50 41–49. 10.1016/j.atmosenv.2012.01.005

[B8] Bowers R. M. Sullivan A. P. Costello E. K. Collett J. L. Knight R. Fierer N. (2011). Sources of bacteria in outdoor air across cities in the midwestern United States. *Appl. Environ. Microbiol.* 77 6350–6356. 10.1128/AEM.05498-5411 21803902 PMC3187178

[B9] Brodie E. L. DeSantis T. Z. Parker J. P. M. Zubietta I. X. Piceno Y. M. Andersen G. L. (2007). Urban aerosols harbor diverse and dynamic bacterial populations. *Proc. Natl. Acad. Sci. U.S.A.* 104 299–304. 10.1073/pnas.0608255104 17182744 PMC1713168

[B10] Budhavant K. B. (2014). Chemical composition of snow-water and scavenging ratios over Costal Antarctica. *Aerosol. Air Qual. Res.* 14 666–676. 10.4209/aaqr.2013.03.0104

[B11] Burrows S. M. Butler T. Jöckel P. Tost H. Kerkweg A. Pöschl U. (2009). Bacteria in the global atmosphere–Part 2: modeling of emissions and transport between different ecosystems. *Atmos. Chem. Phys.* 9 9281–9297.

[B12] Cáliz J. Triadó-Margarit X. Camarero L. Casamayor E. O. (2018). A long-term survey unveils strong seasonal patterns in the airborne microbiome coupled to general and regional atmospheric circulations. *Proc. Natl. Acad. Sci. U.S.A* 115 12229–12234. 10.1073/pnas.1812826115 30420511 PMC6275539

[B13] Carotenuto F. Georgiadis T. Gioli B. Leyronas C. Morris C. E. Nardino M. (2017). Measurements and modeling of surface–atmosphere exchange of microorganisms in Mediterranean grassland. *Atmos. Chem. Phys.* 17 14919–14936. 10.5194/acp-17-14919-12017

[B14] Chemidlin Prévost-Bouré N. Christen R. Dequiedt S. Mougel C. Lelièvre M. Jolivet C. (2011). Validation and application of a PCR primer set to quantify fungal communities in the soil environment by real-time quantitative PCR. *PLoS One* 6:e24166. 10.1371/journal.pone.0024166 21931659 PMC3169588

[B15] China S. Burrows S. M. Wang B. Harder T. H. Weis J. Tanarhte M. (2018). Fungal spores as a source of sodium salt particles in the Amazon basin. *Nat. Commun.* 9:4793. 10.1038/s41467-018-07066-7064 30451836 PMC6242827

[B16] Christner B. C. Morris C. E. Foreman C. M. Cai R. Sands D. C. (2008). Ubiquity of biological ice nucleators in snowfall. *Science* 319 1214–1214. 10.1126/science.1149757 18309078

[B17] Chuvochina M. S. Marie D. Chevaillier S. Petit J.-R. Normand P. Alekhina I. A. (2011). Community variability of bacteria in alpine snow (mont blanc) containing saharan dust deposition and their snow colonisation potential. *Microbes Environ.* 26 237–247. 10.1264/jsme2.ME11116 21666389

[B18] Cotton W. R. Alexander G. D. Hertenstein R. Walko R. L. McAnelly R. L. Nicholls M. (1995). Cloud venting — A review and some new global annual estimates. *Earth Sci. Rev.* 39 169–206. 10.1016/0012-8252(95)00007-0

[B19] Cowan D. A. Chown S. L. Convey P. Tuffin M. Hughes K. Pointing S. (2011). Non-indigenous microorganisms in the Antarctic: assessing the risks. *Trends Microbiol.* 19 540–548. 10.1016/j.tim.2011.07.008 21893414

[B20] De Wekker S. F. J. Kossmann M. (2015). Convective boundary layer heights over mountainous terrain—a review of concepts. *Front. Earth Sci.* 3:77 10.3389/feart.2015.00077

[B21] Dillon J. G. Fishbain S. Miller S. R. Bebout B. M. Habicht K. S. Webb S. M. (2007). High rates of sulfate reduction in a low-sulfate hot spring microbial mat are driven by a low level of diversity of sulfate-respiring microorganisms. *Appl. Environ. Microbiol.* 73 5218–5226. 10.1128/AEM.00357-357 17575000 PMC1950965

[B22] Domine F. Albert M. Huthwelker T. Jacobi H.-W. Kokhanovsky A. A. Lehning M. (2008). Snow physics as relevant to snow photochemistry. *Atmos. Chem. Phys.* 8 171–208. 10.5194/acp-8-171-2008

[B23] Domine F. Shepson P. B. (2002). Air-snow interactions and atmospheric chemistry. *Science* 297 1506–1510. 10.1126/science.1074610 12202818

[B24] Dommergue A. Amato P. Tignat-Perrier R. Magand O. Thollot A. Joly M. (2019). Methods to investigate the global atmospheric microbiome. *Front. Microbiol.* 10:243. 10.3389/fmicb.2019.00243 30967843 PMC6394204

[B25] Els N. Larose C. Baumann-Stanzer K. Tignat-Perrier R. Keuschnig C. Vogel T. M. (2019). Microbial composition in seasonal time series of free tropospheric air and precipitation reveals community separation. *Aerobiologia* 35 671–701. 10.1007/s10453-019-09606-x

[B26] Evans S. E. Dueker M. E. Logan J. R. Weathers K. C. (2019). The biology of fog: results from coastal Maine and Namib desert reveal common drivers of fog microbial composition. *Sci. Total Environ.* 647 1547–1556. 10.1016/j.scitotenv.2018.08.045 30180359

[B27] Fierer N. Liu Z. Rodríguez-Hernández M. Knight R. Henn M. Hernandez M. T. (2008). Short-Term temporal variability in airborne bacterial and fungal populations. *Appl. Environ. Microbiol.* 74 200–207. 10.1128/AEM.01467-1467 17981945 PMC2223228

[B28] Forbes P. B. C. Garland R. M. (2016). “Chapter 4 - Outdoor Air Pollution,” in *Comprehensive Analytical Chemistry The Quality of Air*, eds de la Guardia M. Armenta S. (Amsterdam: Elsevier), 73–96. 10.1016/bs.coac.2016.02.004

[B29] Franz T. P. Eisenreich S. J. (1998). Snow scavenging of polychlorinated biphenyls and polycyclic aromatic hydrocarbons in minnesota. *Environ. Sci. Technol.* 32 1771–1778. 10.1021/es970601z

[B30] Franzetti A. Gandolfi I. Gaspari E. Ambrosini R. Bestetti G. (2011). Seasonal variability of bacteria in fine and coarse urban air particulate matter. *Appl. Microbiol. Biotechnol.* 90 745–753. 10.1007/s00253-010-3048-3047 21184061

[B31] Gallée H. Guyomarc’h G. Brun E. (2001). Impact of snow drift on the antarctic ice sheet surface mass balance: possible sensitivity to snow-surface properties. *Boundary Layer Meteorol.* 99 1–19. 10.1023/A:1018776422809

[B32] Gandolfi I. Bertolini V. Bestetti G. Ambrosini R. Innocente E. Rampazzo G. (2015). Spatio-temporal variability of airborne bacterial communities and their correlation with particulate matter chemical composition across two urban areas. *Appl. Microbiol. Biotechnol.* 99 4867–4877. 10.1007/s00253-014-6348-6345 25592734

[B33] Grannas A. M. Jones A. E. Dibb J. Ammann M. Anastasio C. Beine H. J. (2007). An overview of snow photochemistry: evidence, mechanisms and impacts. *Atmos. Chem. Phys.* 7 4329–4373. 10.5194/acp-7-4329-2007

[B34] Greilinger M. Schauer G. Baumann-Stanzer K. Skomorowski P. Schöner W. Kasper-Giebl A. (2018). Contribution of saharan dust to ion deposition loads of high alpine snow packs in Austria (1987–2017). *Front. Earth Sci.* 6:126 10.3389/feart.2018.00126

[B35] Greilinger M. Schöner W. Winiwarter W. Kasper-Giebl A. (2016). Temporal changes of inorganic ion deposition in the seasonal snow cover for the Austrian Alps (1983–2014). *Atmos. Environ.* 132 141–152. 10.1016/j.atmosenv.2016.02.040

[B36] Greilinger M. Zbiral J. Kasper-Giebl A. (2019). Desert dust contribution to PM10 loads in Styria (Southern Austria) and impact on exceedance of limit values from 2013–2018. *Appl. Sci.* 9:2265 10.3390/app9112265

[B37] Harder S. Warren S. G. Charlson R. J. (2000). Sulfate in air and snow at the South Pole: implications for transport and deposition at sites with low snow accumulation. *J. Geophys. Res. Atmos.* 105 22825–22832. 10.1029/2000JD900351

[B38] Harding T. Jungblut A. D. Lovejoy C. Vincent W. F. (2011). Microbes in high arctic snow and implications for the cold biosphere. *Appl. Environ. Microbiol.* 77 3234–3243. 10.1128/AEM.02611-2610 21460114 PMC3126466

[B39] Hauptmann A. L. Stibal M. Baelum J. Sicheritz-Pontén T. Brunak S. Bowman J. S. (2014). Bacterial diversity in snow on North Pole ice floes. *Extremophiles* 18 945–951. 10.1007/s00792-014-0660-y 24951969 PMC4196135

[B40] Hell K. Edwards A. Zarsky J. Podmirseg S. M. Girdwood S. Pachebat J. A. (2013). The dynamic bacterial communities of a melting High Arctic glacier snowpack. *ISME J.* 7 1814–1826. 10.1038/ismej.2013.51 23552623 PMC3749494

[B41] Herrmann H. Exner M. Zellner R. (1994). Reactivity trends in reactions of the nitrate radical (NO3) with inorganic and organic cloudwater constituents. *Geochim. Cosmochim. Acta* 58 3239–3244. 10.1016/0016-7037(94)90051-90055

[B42] Kalthoff N. Träumner K. Adler B. Späth S. Behrendt A. Wieser A. (2013). Dry and moist convection in the boundary layer over the Black Forest - A combined analysis of in situ and remote sensing data. *Meteorol. Zeitschrift* 22 445–461. 10.1127/0941-2948/2013/0417

[B43] Kennedy K. Hall M. W. Lynch M. D. J. Moreno-Hagelsieb G. Neufeld J. D. (2014). Evaluating bias of Illumina-based bacterial 16S rRNA gene profiles. *Appl. Environ. Microbiol.* 80 5717–5722. 10.1128/AEM.01451-1414 25002428 PMC4178620

[B44] Klindworth A. Pruesse E. Schweer T. Peplies J. Quast C. Horn M. (2013). Evaluation of general 16S ribosomal RNA gene PCR primers for classical and next-generation sequencing-based diversity studies. *Nucleic Acids Res.* 41:e1. 10.1093/nar/gks808 22933715 PMC3592464

[B45] Larose C. Berger S. Ferrari C. Navarro E. Dommergue A. Schneider D. (2010). Microbial sequences retrieved from environmental samples from seasonal Arctic snow and meltwater from Svalbard. *Norway. Extremophiles* 14 205–212. 10.1007/s00792-009-0299-2 20066448

[B46] Laughlin R. J. Stevens R. J. Müller C. Watson C. J. (2008). Evidence that fungi can oxidize NH4+ to NO3- in a grassland soil. *Eur. J. Soil Sci.* 59 285–291. 10.1111/j.1365-2389.2007.00995.x

[B47] Lazzaro A. Hilfiker D. Zeyer J. (2015). Structures of microbial communities in alpine soils: seasonal and elevational effects. *Front. Microbiol.* 6:1330. 10.3389/fmicb.2015.01330 26635785 PMC4660872

[B48] Lehning M. Bartelt P. Brown B. Russi T. Stöckli U. Zimmerli M. (1999). snowpack model calculations for avalanche warning based upon a new network of weather and snow stations. *Cold Reg. Sci. Technol.* 30 145–157. 10.1016/S0165-232X(99)00022-21

[B49] Li Z. Edwards R. Mosley-Thompson E. Wang F. Dong Z. You X. (2006). Seasonal variability of ionic concentrations in surface snow and elution processes in snow–firn packs at the PGPI site on Ürümqi glacier No. 1, eastern Tien Shan, China. *Ann. Glaciol.* 43 250–256. 10.3189/172756406781812069

[B50] Lotteraner C. Piringer M. (2016). Mixing-Height time series from operational ceilometer aerosol-layer heights. *Boundary Layer Meteorol.* 161 265–287. 10.1007/s10546-016-0169-162

[B51] Maccario L. Carpenter S. D. Deming J. W. Vogel T. M. Larose C. (2019). Sources and selection of snow-specific microbial communities in a Greenlandic sea ice snow cover. *Sci. Rep.* 9:2290. 10.1038/s41598-019-38744-y 30783153 PMC6381142

[B52] Maccario L. Vogel T. M. Larose C. (2014). Potential drivers of microbial community structure and function in Arctic spring snow. *Front. Microbiol.* 5:413. 10.3389/fmicb.2014.00413 25147550 PMC4124603

[B53] Margesin R. Miteva V. (2011). Diversity and ecology of psychrophilic microorganisms. *Res. Microbiol.* 162 346–361. 10.1016/j.resmic.2010.12.004 21187146

[B54] Maria S. F. Russell L. M. (2005). Organic and inorganic aerosol below-cloud scavenging by suburban New Jersey precipitation. *Environ. Sci. Technol.* 39 4793–4800. 10.1021/es0491679 16053076

[B55] Martinez Arbizu P. (2017). *pairwiseAdonis: Pairwise Multilevel Comparison Using Adonis. R Package Version 0.0.1.*

[B56] Masella A. P. Bartram A. K. Truszkowski J. M. Brown D. G. Neufeld J. D. (2012). PANDAseq: paired-end assembler for illumina sequences. *BMC Bioinformatics* 13:31. 10.1186/1471-2105-13-31 22333067 PMC3471323

[B57] McMurdie P. J. Holmes S. (2013). phyloseq: an R Package for reproducible interactive analysis and graphics of microbiome census data. *PLoS One* 8:e61217. 10.1371/journal.pone.0061217 23630581 PMC3632530

[B58] Meola M. Lazzaro A. Zeyer J. (2015). Bacterial composition and survival on sahara dust particles transported to the European Alps. *Front. Microbiol.* 6:1454. 10.3389/fmicb.2015.01454 26733988 PMC4686684

[B59] Mizrahi-Man O. Davenport E. R. Gilad Y. (2013). Taxonomic classification of bacterial 16S rRNA genes using short sequencing reads: evaluation of effective study designs. *PLoS One* 8:e53608. 10.1371/journal.pone.0053608 23308262 PMC3538547

[B60] Möhler O. Demott P. J. Vali G. Levin Z. (2007). Microbiology and atmospheric processes: the role of biological particles in cloud physics. *Biogeosciences* 4 1059–1071.

[B61] Monteil C. L. Bardin M. Morris C. E. (2014). Features of air masses associated with the deposition of *Pseudomonas syringae* and Botrytis cinerea by rain and snowfall. *ISME J.* 8 2290–2304. 10.1038/ismej.2014.55 24722630 PMC4992071

[B62] Oksanen J. Blanchet F. G. Friendly M. Kindt R. Legendre P. McGlinn D. (2018). *vegan: Community Ecology Package.* Available online at: https://CRAN.R-project.org/package=vegan (accessed May 4, 2018).

[B63] Olefs M. Baumgartner D. J. Obleitner F. Bichler C. Foelsche U. Pietsch H. (2016). The Austrian radiation monitoring network ARAD – best practice and added value. *Atmos. Meas. Tech.* 9 1513–1531. 10.5194/amt-9-1513-2016

[B64] Øvreås L. Torsvik V. (1998). Microbial diversity and community structure in two different agricultural soil communities. *Microb. Ecol.* 36 303–315. 10.1007/s002489900117 9852510

[B65] Paramonov M. Grönholm T. Virkkula A. (2011). Below-cloud scavenging of aerosol particles by snow at an urban site in Finland. *Boreal Environ. Res.* 16 304–320.

[B66] Pedgley D. E. (1991). “Aerobiology: the atmosphere as a source and sink for microbes,” in *Microbial Ecology of Leaves Brock/Springer Series in Contemporary Bioscience*, eds Andrews J. H. Hirano S. S. (New York, NY: Springer), 43–59.

[B67] Perrone M. G. Gualtieri M. Ferrero L. Porto C. L. Udisti R. Bolzacchini E. (2010). Seasonal variations in chemical composition and in vitro biological effects of fine PM from Milan. *Chemosphere* 78 1368–1377. 10.1016/j.chemosphere.2009.12.071 20123145

[B68] Peter H. Hörtnagl P. Reche I. Sommaruga R. (2014). Bacterial diversity and composition during rain events with and without Saharan dust influence reaching a high mountain lake in the Alps. *Environ. Microbiol. Rep.* 6 618–624. 10.1111/1758-2229.12175 25756115 PMC4733657

[B69] Pickersgill D. A. Wehking J. Paulsen H. Thines E. Pöschl U. Fröhlich-Nowoisky J. (2017). Lifestyle dependent occurrence of airborne fungi. *Biogeosci. Discuss.* 2017 1–20. 10.5194/bg-2017-2452

[B70] Pio C. A. Legrand M. Oliveira T. Afonso J. Santos C. Caseiro A. (2007). Climatology of aerosol composition (organic versus inorganic) at nonurban sites on a west-east transect across Europe. *J. Geophys. Res.* 112:D23S02 10.1029/2006JD008038

[B71] Polymenakou P. N. (2012). Atmosphere: a source of pathogenic or beneficial microbes? *Atmosphere* 3 87–102. 10.3390/atmos3010087

[B72] Polymenakou P. N. Mandalakis M. (2013). Assessing the short-term variability of bacterial composition in background aerosols of the Eastern Mediterranean during a rapid change of meteorological conditions. *Aerobiologia* 29 429–441. 10.1007/s10453-013-9295-9291

[B73] R Core Team (2015). *R: A Language and Environment for Statistical Computing. R: A Language and Environment for Statistical Computing.* Available online at: https://www.R-project.org (accessed October 14, 2015).

[B74] Šantl-Temkiv T. Gosewinkel U. Starnawski P. Lever M. Finster K. (2018). Aeolian dispersal of bacteria in southwest Greenland: their sources, abundance, diversity and physiological states. *FEMS Microbiol. Ecol.* 94:fiy031. 10.1093/femsec/fiy031 29481623

[B75] Segawa T. Miyamoto K. Ushida K. Agata K. Okada N. Kohshima S. (2005). Seasonal change in bacterial flora and biomass in mountain snow from the Tateyama Mountains, Japan, analyzed by 16S rRNA gene sequencing and real-time PCR. *Appl. Environ. Microbiol.* 71 123–130. 10.1128/AEM.71.1.123-130.2005 15640179 PMC544271

[B76] Smith D. J. Griffin Dale W. Jaffe Daniel A. (2011). The high life: transport of microbes in the atmosphere. *Eos Trans. Am. Geophys. Union* 92 249–250. 10.1029/2011EO300001

[B77] Spear J. R. Honeyman A. S. Day M. L. (2018). Fresh snowfall microbiology and chemistry are driven by geography in storm-tracked events. *PeerJ* 6:e5961. 10.1101/300772 30498637 PMC6252068

[B78] Squizzato S. Masiol M. Brunelli A. Pistollato S. Tarabotti E. Rampazzo G. (2013). Factors determining the formation of secondary inorganic aerosol: a case study in the Po Valley (Italy). *Atmos. Chem. Phys.* 13 1927–1939. 10.5194/acp-13-1927-2013

[B79] Taylor D. L. Walters W. A. Lennon N. J. Bochicchio J. Krohn A. Caporaso J. G. (2016). Accurate estimation of fungal diversity and abundance through improved lineage-specific primers optimized for illumina amplicon sequencing. *Appl. Environ. Microbiol.* 82 7217–7226. 10.1128/AEM.02576-2516 27736792 PMC5118932

[B80] Tignat-Perrier R. Dommergue A. Thollot A. Keuschnig C. Magand O. Vogel T. M. (2019). Global airborne microbial communities controlled by surrounding landscapes and wind conditions. *Sci. Rep.* 9:14441. 10.1038/s41598-019-51073-51074 31595018 PMC6783533

[B81] Triadó-Margarit X. Caliz J. Reche I. Casamayor E. O. (2019). High similarity in bacterial bioaerosol compositions between the free troposphere and atmospheric depositions collected at high-elevation mountains. *Atmos. Environ.* 203 79–86. 10.1016/j.atmosenv.2019.01.038

[B82] Wang Q. Garrity G. M. Tiedje J. M. Cole J. R. (2007). Naive Bayesian classifier for rapid assignment of rRNA sequences into the new bacterial taxonomy. *Appl. Environ. Microbiol.* 73 5261–5267. 10.1128/AEM.00062-67 17586664 PMC1950982

[B83] Wang X. Zhang L. Moran M. D. (2014). Development of a new semi-empirical parameterization for below-cloud scavenging of size-resolved aerosol particles by both rain and snow. *Geosci. Model Dev.* 7 799–819. 10.5194/gmd-7-799-2014

[B84] Weil T. De Filippo C. Albanese D. Donati C. Pindo M. Pavarini L. (2017). Legal immigrants: invasion of alien microbial communities during winter occurring desert dust storms. *Microbiome* 5:32. 10.1186/s40168-017-0249-247 28283029 PMC5345179

[B85] Weiss S. Xu Z. Z. Peddada S. Amir A. Bittinger K. Gonzalez A. (2017). Normalization and microbial differential abundance strategies depend upon data characteristics. *Microbiome* 5:27. 10.1186/s40168-017-0237-y 28253908 PMC5335496

[B86] Wickham H. (2009). *ggplot2: Elegant Graphics for Data Analysis.* New-York, NY: Springer-Verlag.

[B87] Woo C. An C. Xu S. Yi S.-M. Yamamoto N. (2018). Taxonomic diversity of fungi deposited from the atmosphere. *ISME J.* 12:2051. 10.1038/s41396-018-0160-167 29849168 PMC6051994

[B88] Xia Y. Conen F. Alewell C. (2013). Total bacterial number concentration in free tropospheric air above the Alps. *Aerobiologia* 29 153–159. 10.1007/s10453-012-9259-x

[B89] Yamamoto N. Bibby K. Qian J. Hospodsky D. Rismani-Yazdi H. Nazaroff W. W. (2012). Particle-size distributions and seasonal diversity of allergenic and pathogenic fungi in outdoor air. *ISME J.* 6 1801–1811. 10.1038/ismej.2012.30 22476354 PMC3446800

[B90] Zhang H. Zhou X. Zou J. Wang W. Xue L. Ding Q. (2018). A review on the methods for observing the substance and energy exchange between atmosphere boundary layer and free troposphere. *Atmosphere* 9:460 10.3390/atmos9120460

[B91] Zhang L. Wang X. Moran M. D. Feng J. (2013). Review and uncertainty assessment of size-resolved scavenging coefficient formulations for below-cloud snow scavenging of atmospheric aerosols. *Atmos. Chem. Phys.* 13 10005–10025. 10.5194/acp-13-10005-12013

