# Supplementary material for: Comparison of Bacterial and Fungal Composition and Their Chemical Interaction in Free Tropospheric Air and Snow Over an Entire Winter Season at Mount Sonnblick, Austria
Source: Front Microbiol. 2020 May 20;11:980. doi: 10.3389/fmicb.2020.00980 (PMC7251065; doi:10.3389/fmicb.2020.00980)
Supplement: Supplementary file 1 [file Data_Sheet_1.docx]

***Supplementary Material***

**(A)**

**(B)**


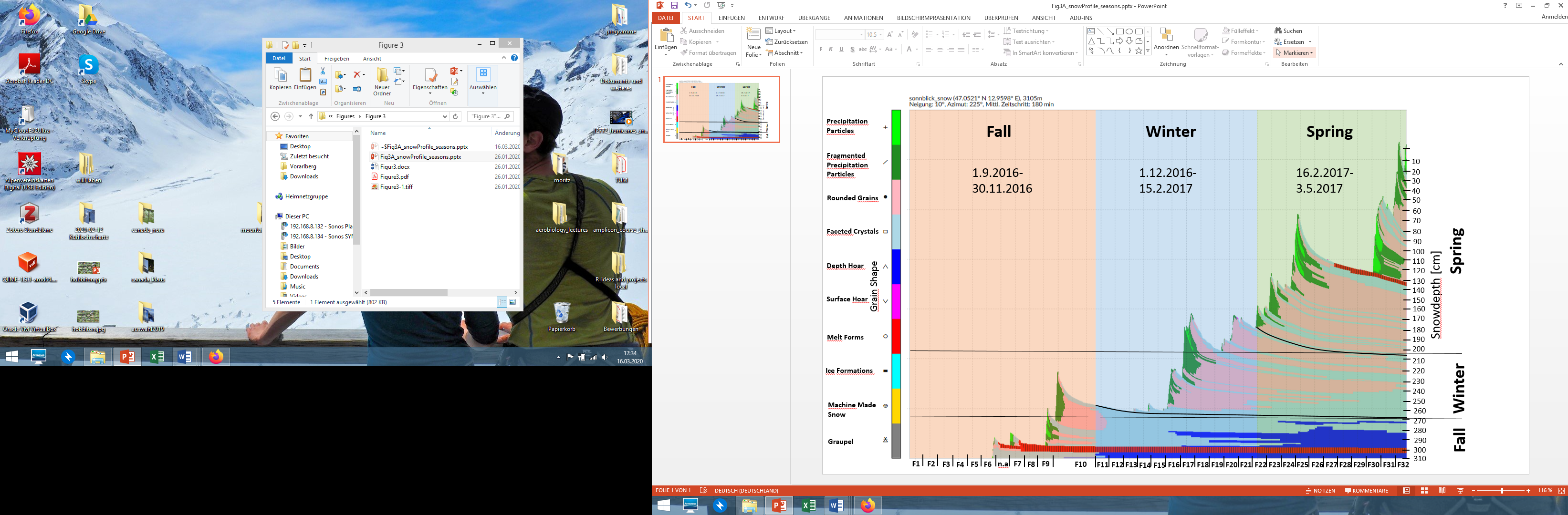

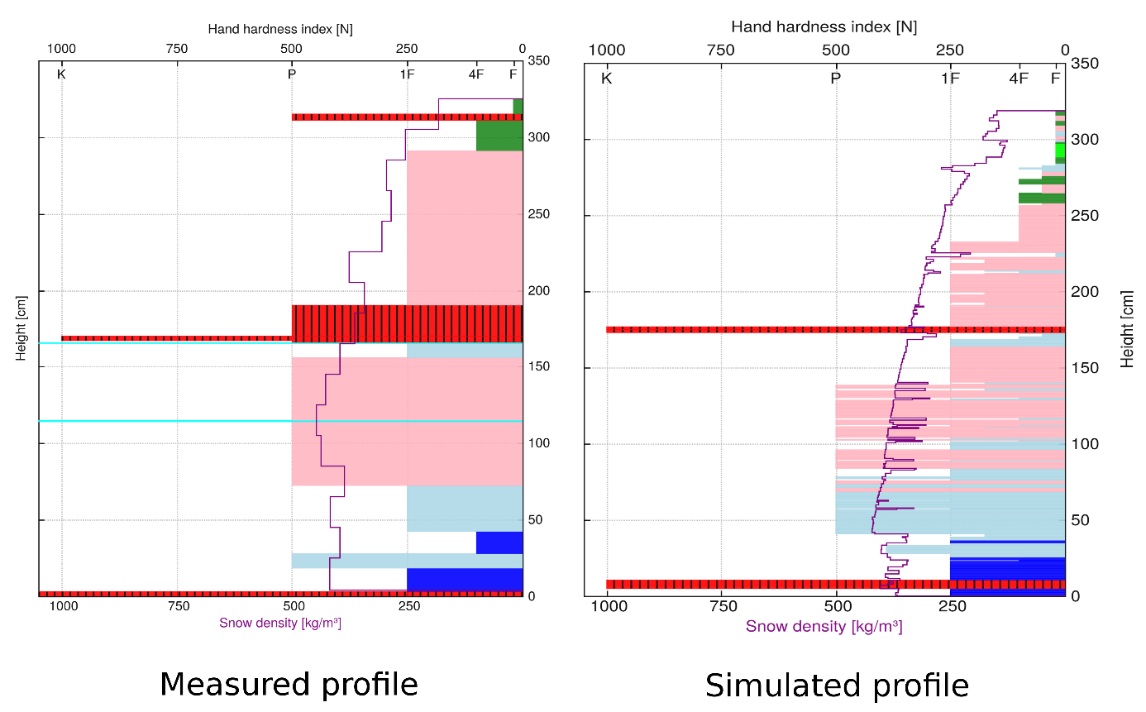


**Faceted Crystals**

**Depth Hoar**

**Surface Hoar**

**Melt Forms**

**Ice Formations**

**Rounded Grains**

**Fragmented Precipitation Particles**

**Precipitation Particles**

**SI Figure 1:** A) Manually recorded profile in the field, B) profile simulated by SNOWPACK


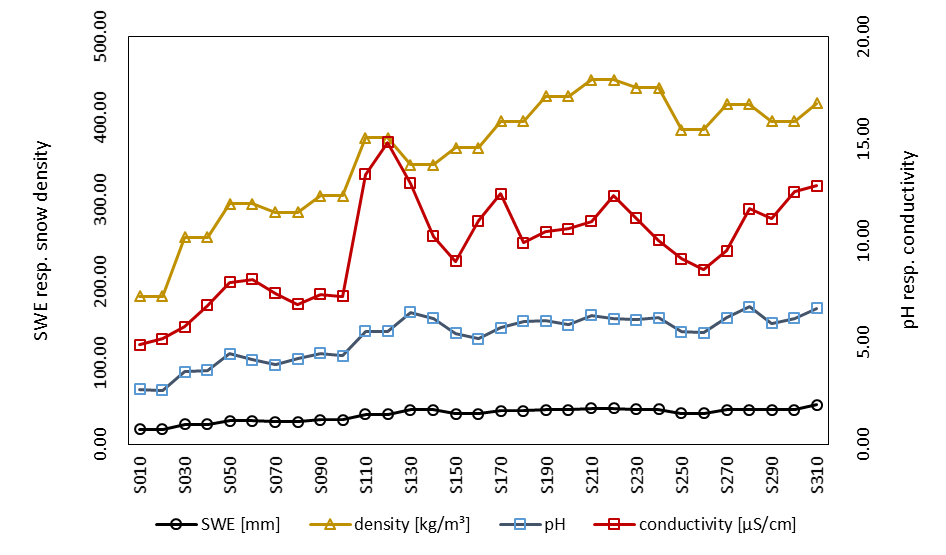

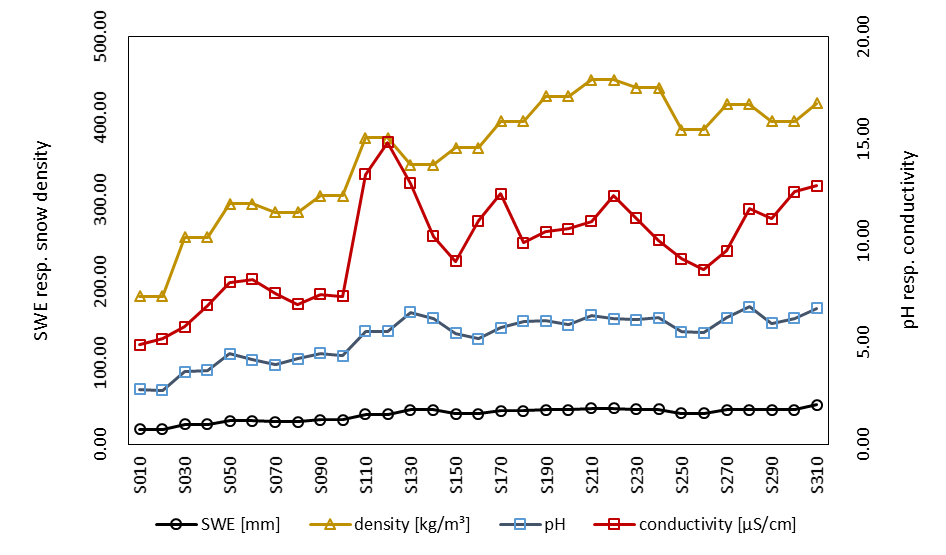


**SI Figure 2:** Physical properties of the snow profile

**
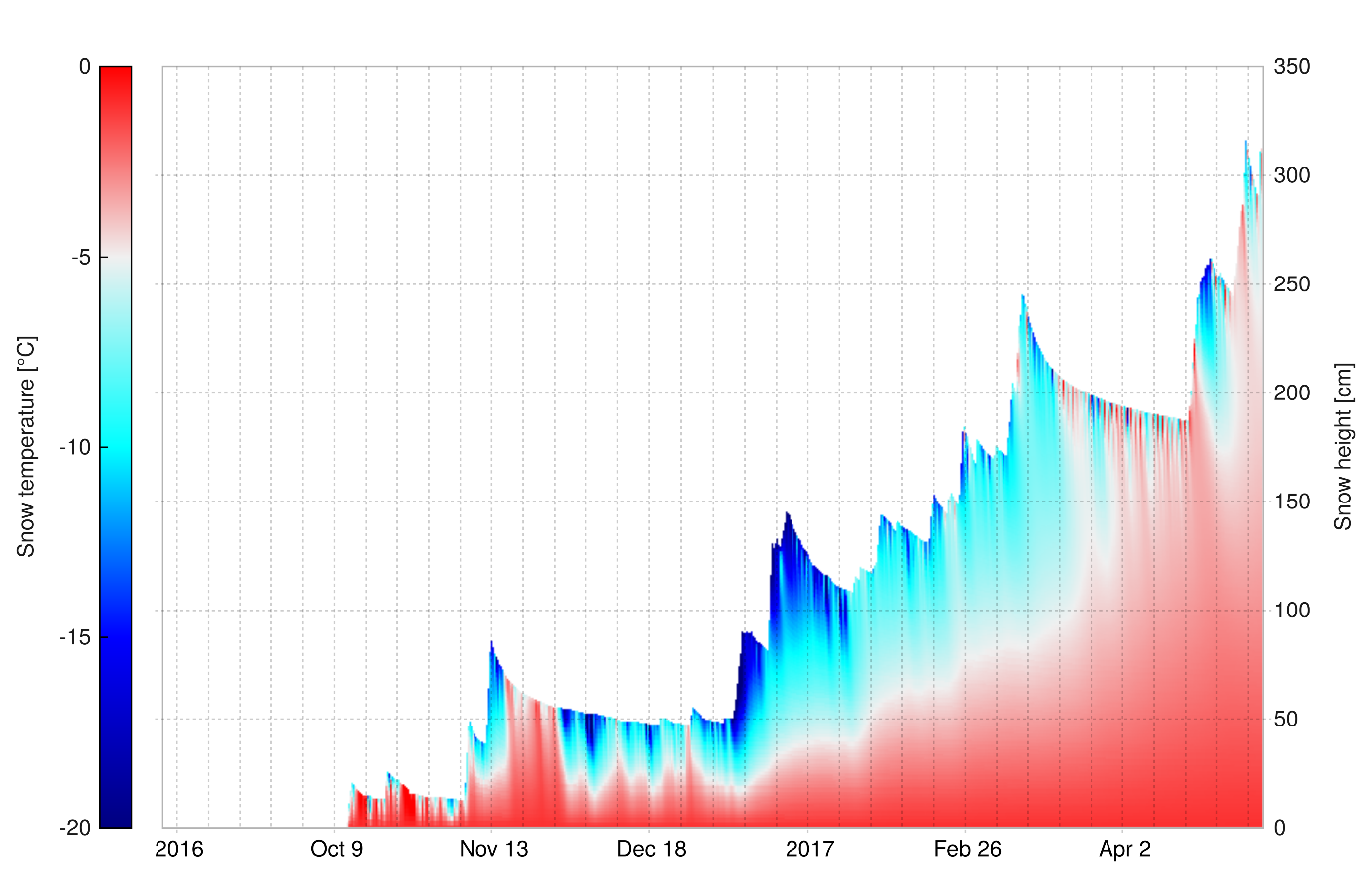

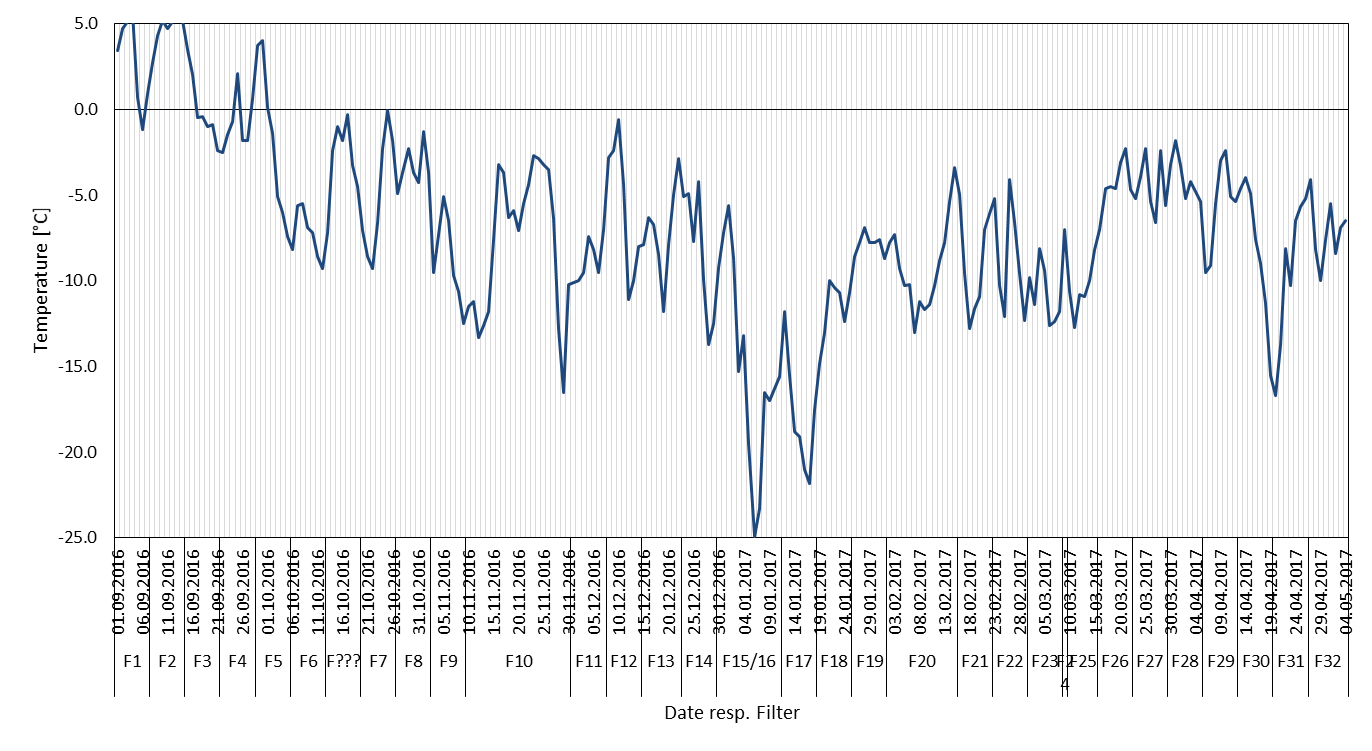
**

**SI Figure 4:** Simulated snow temperature in the snowpack

**SI Figure 3:** Daily mean air temperature [°C] measured at the automatic snow gauge (TAWES #11343) during the sampling period

| **AIR & SNOW** | 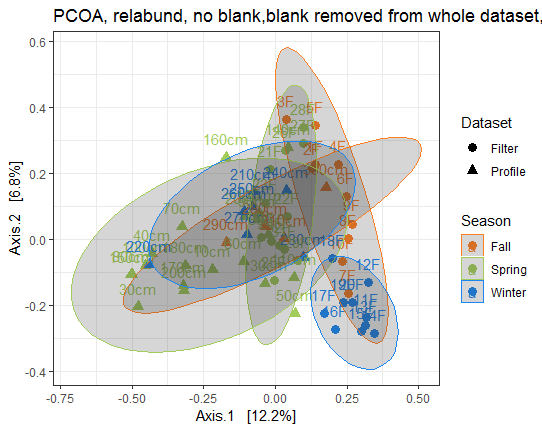  **(A)** | 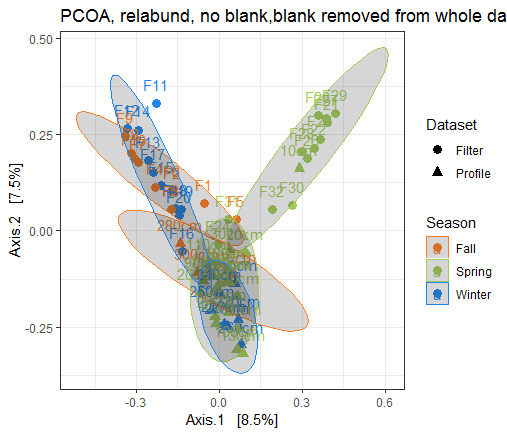  **(B)** |
| --- | --- | --- |

**SI Figure 5:** PCoA of air and snow dataset coloured by seasons, A) bacteria, B) fungi

| **Bacteria** | **Fungi** |
| --- | --- |
| 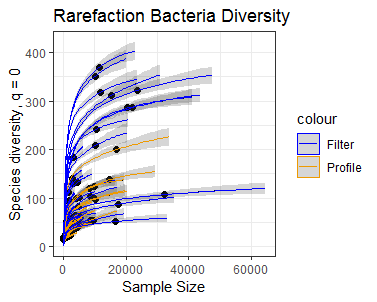  **(A)** | 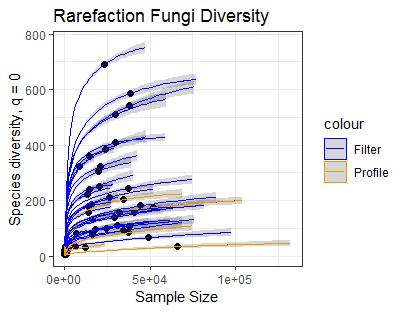 |
| 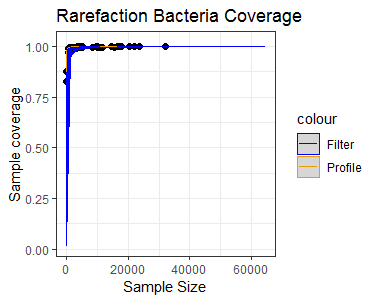  **(B)** | 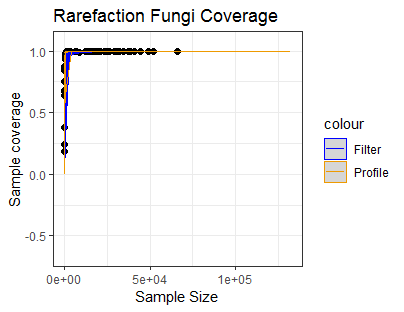 |

**SI Figure 6:** Rarefaction curves for A) bacteria and B) fungi

**SI Table 1:** Sequence statistics for the analysed dataset

|  | **Bacteria** | **Fungi** |
| --- | --- | --- |
| **#mean raw sequences per sample** | 20153+-11280 | 20899+-21582 |
| **# genera raw dataset** | 1566 | 3127 |
| **# genera after blank removal** | 1058 | 1902 |

**SI Table 2:** ANOSIM and ADONIS for air filter vs. snow profile

| **Bacteria** | | | **Fungi** | | |
| --- | --- | --- | --- | --- | --- |
| **ANOSIM** | **R** | **p** | **ANOSIM** | **R** | **p** |
| Filter~Profile | 0.325 | 0.001** | Filter~Profile | 0.287 | 0.001** |
| **(bray)** |  |  | **(bray)** |  |  |
| **ADONIS** | **Df** | **F-Model** | **ADONIS** | **Df** | **F-Model** |
| Filter~Profile | 1 | 4.6579 | Filter~Profile | 1 | 3.8763 |
| **(bray)** | **R²** | **p** | **(bray)** | **R²** | **p** |
|  | 0.07094 | 0.001** |  | 0.05796 | 0.001** |

**SI Table 3:** PERMANOVA by season and dataset

| **Bacteria** | | | | | | **Fungi** | | | | | |
| --- | --- | --- | --- | --- | --- | --- | --- | --- | --- | --- | --- |
| **pairs** | **Df** | **F-Model** | **R2** | **p-value** | **p.adj** | **pairs** | **Df** | **F-Model** | **R2** | **p-value** | **p.adj** |
| **ProfileSpring vs**  **FilterFall** | 29 | 4.32 | 0.13 | 0.001 | **0.015** | **ProfileSpring vs FilterFall** | 29 | 3.11 | 0.10 | 0.001 | **0.015** |
| **ProfileSpring vs**  **FilterWinter** | 29 | 5.63 | 0.16 | 0.001 | **0.015** | **ProfileSpring vs FilterWinter** | 29 | 3.26 | 0.10 | 0.001 | **0.015** |
| **ProfileSpring vs**  **ProfileWinter** | 26 | 1.39 | 0.05 | 0.063 | 0.945 | **ProfileSpring vs ProfileWinter** | 26 | 0.96 | 0.03 | 0.562 | 1.000 |
| **ProfileSpring vs**  **FilterSpring** | 31 | 2.59 | 0.07 | 0.001 | **0.015** | **ProfileSpring vs FilterSpring** | 31 | 3.50 | 0.10 | 0.001 | **0.015** |
| **ProfileSpring vs**  **ProfileFall** | 23 | 1.85 | 0.07 | 0.006 | 0.090 | **ProfileSpring vs ProfileFall** | 23 | 1.39 | 0.05 | 0.018 | 0.270 |
| **FilterFall vs**  **FilterWinter** | 19 | 3.13 | 0.14 | 0.001 | **0.015** | **FilterFall vs**  **FilterWinter** | 19 | 2.69 | 0.12 | 0.001 | **0.015** |
| **FilterFall vs**  **ProfileWinter** | 16 | 2.49 | 0.14 | 0.001 | **0.015** | **FilterFall vs**  **ProfileWinter** | 16 | 2.81 | 0.15 | 0.001 | **0.015** |
| **FilterFall vs**  **FilterSpring** | 21 | 1.91 | 0.08 | 0.003 | **0.045** | **FilterFall vs**  **FilterSpring** | 21 | 4.75 | 0.19 | 0.001 | **0.015** |
| **FilterFall vs**  **ProfileFall** | 13 | 2.15 | 0.15 | 0.005 | 0.075 | **FilterFall vs**  **ProfileFall** | 13 | 2.22 | 0.15 | 0.001 | **0.015** |
| **FilterWinter vs**  **ProfileWinter** | 16 | 3.54 | 0.19 | 0.001 | **0.015** | **FilterWinter vs**  **ProfileWinter** | 16 | 3.11 | 0.17 | 0.001 | **0.015** |
| **FilterWinter vs**  **FilterSpring** | 21 | 3.45 | 0.14 | 0.001 | **0.015** | **FilterWinter vs**  **FilterSpring** | 21 | 5.13 | 0.20 | 0.001 | **0.015** |
| **FilterWinter vs**  **ProfileFall** | 13 | 3.15 | 0.20 | 0.002 | **0.030** | **FilterWinter vs**  **ProfileFall** | 13 | 2.46 | 0.17 | 0.002 | **0.030** |
| **ProfileWinter vs**  **FilterSpring** | 18 | 1.13 | 0.06 | 0.208 | 1.000 | **ProfileWinter vs FilterSpring** | 18 | 3.12 | 0.15 | 0.001 | **0.015** |
| **ProfileWinter vs**  **ProfileFall** | 10 | 1.29 | 0.12 | 0.080 | 1.000 | **ProfileWinter vs ProfileFall** | 10 | 1.15 | 0.11 | 0.199 | 1.000 |
| **FilterSpring vs**  **ProfileFall** | 15 | 1.37 | 0.08 | 0.050 | 0.750 | **FilterSpring vs**  **ProfileFall** | 15 | 2.71 | 0.16 | 0.002 | **0.030** |

**SI Table 4:** Wilcoxon alpha diversity test statistics

| **Bacteria** | | | **Fungi** | | |
| --- | --- | --- | --- | --- | --- |
|  | **W** | **P** |  | **W** | **P** |
| Chao1 | 789.5 | **<0.0001** | Chao1 | 1002 | **<0.0001** |
| Shannon | 759 | **0.0002** | Shannon | 951 | **<0.0001** |

**SI Table 5:** Pairwise wilcox test per season and datatype for bacteria, not p-adjusted values in ()

| **Bacteria Observed**  **holm p.adjusted (not p.adjusted)** | | | | | |
| --- | --- | --- | --- | --- | --- |
|  | **FilterFall** | **FilterSpring** | **FilterWinter** | **ProfileFall** | **ProfileSpring** |
| **FilterSpring** | **0.04555 (0.0046)** |  |  |  |  |
| **FilterWinter** | **0.03504**  **(0.0032)** | **0.00122**  **(8.7e-05)** |  |  |  |
| **ProfileFall** | 1.0000  (0.1878) | 1.0000  (0.8556) | 0.06520  **(0.0080)** |  |  |
| **ProfileSpring** | **0.01458**  **(0.0012)** | 1.0000  (0.2199) | **0.00027**  **(1.8e-05)** | 1.0000  (0.8160) |  |
| **ProfileWinter** | 0.06520  **(0.0072)** | 1.0000  (0.1757) | **0.01392**  **(0.0011)** | 1.0000  (0.6358) | 1.0000  (0.8680) |
| **Bacteria Chao1**  **holm p.adjusted (not p.adjusted)** | | | | | |
|  | **FilterFall** | **FilterSpring** | **FilterWinter** | **ProfileFall** | **ProfileSpring** |
| **FilterSpring** | **0.00660**  **(0.00060)** |  |  |  |  |
| **FilterWinter** | **0.04676**  **(0.00520)** | **4.5e-05**  **(3.1e-06)** |  |  |  |
| **ProfileFall** | 0.99301  (0.14186) | 1.0000  (0.77033) | 0.6394  **(0.00799)** |  |  |
| **ProfileSpring** | **0.00477**  **(0.00040)** | 1.0000  (0.41360) | **0.00025**  **(1.8e-05)** | 1.0000  (0.78626) |  |
| **ProfileWinter** | **0.01234**  **(0.00123)** | 1.0000  (0.29912) | **0.00267**  **(0.00021)** | 1.0000  (0.64848) | 1.0000  (0.80335) |
| **Bacteria Shannon**  **holm p.adjusted (not p.adjusted)** | | | | | |
|  | **FilterFall** | **FilterSpring** | **FilterWinter** | **ProfileFall** | **ProfileSpring** |
| **FilterSpring** | **0.00071**  **(5.9e-05)** |  |  |  |  |
| **FilterWinter** | 0.18585  **(0.02323)** | **8.0e-05**  **(6.2e-06)** |  |  |  |
| **ProfileFall** | 0.25175  (0.3596) | 1.0000  (0.44615) | **0.07193**  **(0.00799)** |  |  |
| **ProfileSpring** | **1.8e-05**  **(1.3e-06)** | 1.0000  (0.89329) | **2.0e-06**  **(1.3e-07)** | 1.0000  (0.52494) |  |
| **ProfileWinter** | **0.01954**  **(0.00195)** | 1.0000  (0.48242) | **0.00226**  **(0.00021)** | 1.0000  (0.64848) | 1.0000  (0.53324) |

**SI Table 6:** Pairwise wilcox test per season and datatype for fungi, not p-adjusted values in ()

| **Fungi Observed**  **holm p.adjusted (not p.adjusted)** | | | | | |
| --- | --- | --- | --- | --- | --- |
|  | **FilterFall** | **FilterSpring** | **FilterWinter** | **ProfileFall** | **ProfileSpring** |
| **FilterSpring** | 0.06662  **(0.0083)** |  |  |  |  |
| **FilterWinter** | 1.0000  (0.4967) | **0.01358**  **(0.0015)** |  |  |  |
| **ProfileFall** | 0.20140  **(0.0503)** | 1.0000  (0.8706) | 0.08392  **(0.0140)** |  |  |
| **ProfileSpring** | **0.00058**  **(5.2e-05)** | **0.00011**  **(7.5e-06)** | **0.00033**  **(2.7e-05)** | 0.07433  **(0.0106)** |  |
| **ProfileWinter** | **0.00028**  **(2.2e-5)** | **0.00093**  **(9.3e-05)** | **0.00015**  **(1.1e-05)** | 0.08392  **(0.0140)** | 1.0000  (0.6875) |
| **Fungi Chao1**  **holm p.adjusted (not p.adjusted)** | | | | | |
|  | **FilterFall** | **FilterSpring** | **FilterWinter** | **ProfileFall** | **ProfileSpring** |
| **FilterSpring** | 0.21688  **(0.0434)** |  |  |  |  |
| **FilterWinter** | 1.0000  (0.5490) | **0.02029**  **(0.0025)** |  |  |  |
| **ProfileFall** | 0.42517  (0.1063) | 1.0000  (0.7849) | 0.14386  **(0.0240)** |  |  |
| **ProfileSpring** | **5.8e-05**  **(4.5e-06)** | **2.4e-05**  **(1.6e-06)** | **2.4e-05**  **(1.7e-06)** | 0.06316  **(0.0090)** |  |
| **ProfileWinter** | **0.00022**  **(2.2e-05)** | **0.00013**  **(1.2e-05)** | **0.00013**  **(1.1e-05)** | **0.01798**  **(0.0020)** | 1.0000  (0.340) |
| **Fungi Shannon**  **holm p.adjusted (not p.adjusted)** | | | | | |
|  | **FilterFall** | **FilterSpring** | **FilterWinter** | **ProfileFall** | **ProfileSpring** |
| **FilterSpring** | **0.01299**  **(0.00144)** |  |  |  |  |
| **FilterWinter** | 1.0000  (0.84211) | **0.00187**  **(0.00017)** |  |  |  |
| **ProfileFall** | **0.03357**  **(0.00559)** | 1.0000  (0.41176) | **0.02797**  **(0.00400)** |  |  |
| **ProfileSpring** | **1.8e-05**  **(1.3e-06)** | **0.02657**  **(0.00332)** | **3.6e-06**  **(2.4e-07)** | 1.0000  (0.76291) |  |
| **ProfileWinter** | **0.00052**  **(4.3e-05)** | **0.01145**  **(0.00115)** | **0.00014**  **(1.1e-05)** | 1.0000  (0.37363) | 0.59767  (0.11953) |

**SI Table 7:** Ten most abundant bacterial phyla and fungal classes in the complete air and snow dataset

| **Bacteria** | **Air (Filter)** | **Snow (Profile)** | **Fungi** | **Air (Filter)** | **Snow (Profile)** |
| --- | --- | --- | --- | --- | --- |
| **Acidobacteria** | 2.64 | 1.41 | **Agaricomycetes** | 20.79 | 16.85 |
| **Actinobacteria** | 28.09 | 10.50 | **Dothideomycetes** | 19.17 | 27.16 |
| **Bacteriodetes** | 10.11 | 12.69 | **Eurotiomycetes** | 27.34 | 9.75 |
| **Saccharibacteria** | 0.44 | 1.25 | **Lecarnoromycetes** | 0.93 | 10.45 |
| **Chloroflexi** | 1.16 | 1.24 | **Leotiomycetes** | 5.97 | 4.19 |
| **Cyanobacteria** | 2.56 | 4.25 | **Microbotryomycetes** | 1.35 | 7.79 |
| **Deinococcus-Therm** | 0.86 | 0.45 | **Pezizomycetes** | 0.59 | 1.54 |
| **Firmicutes** | 30.29 | 26.91 | **Sordariomycetes** | 16.49 | 7.67 |
| **Gemmatimonadetes** | 0.74 | 0.66 | **Tremellomycetes** | 4.43 | 3.27 |
| **Proteobacteria** | 20.54 | 39.15 | **Ustligainomycotina** | 0.32 | 9.14 |
| **Other** | 2.54 | 1.49 | **Others** | 2.62 | 2.17 |

| **Bacteria**  **(A)** | **Fungi** |
| --- | --- |
| 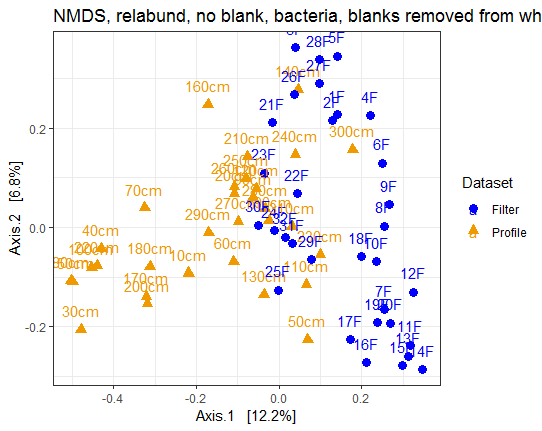 | 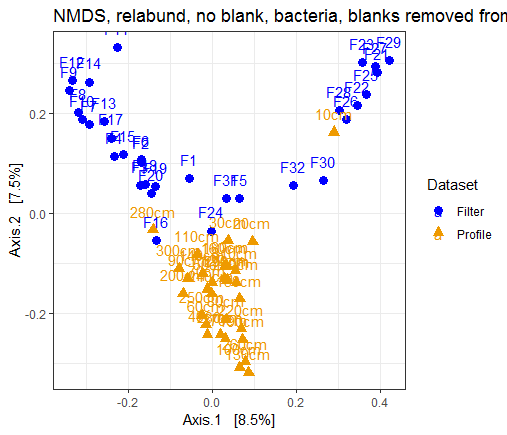 |
| 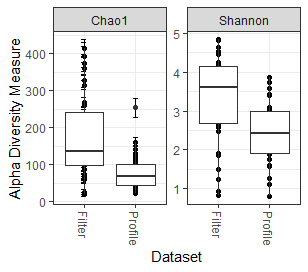  **(B)** | 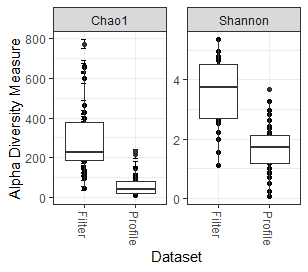 |
| 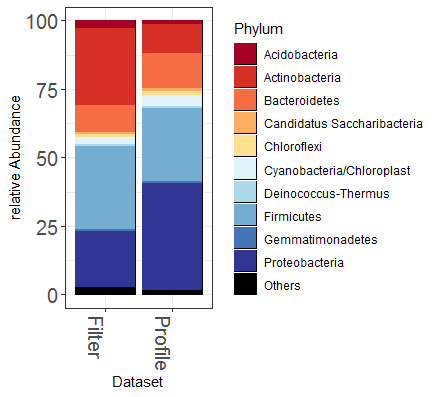  **(C)** | 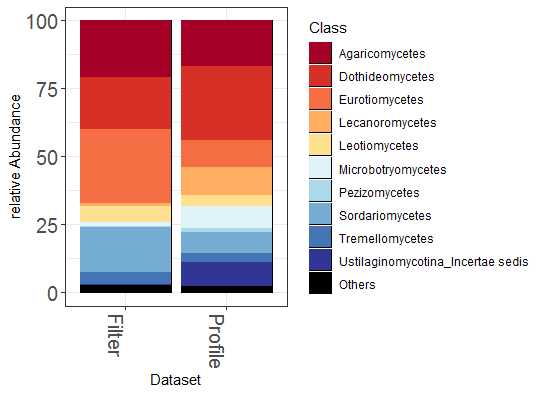 |
| **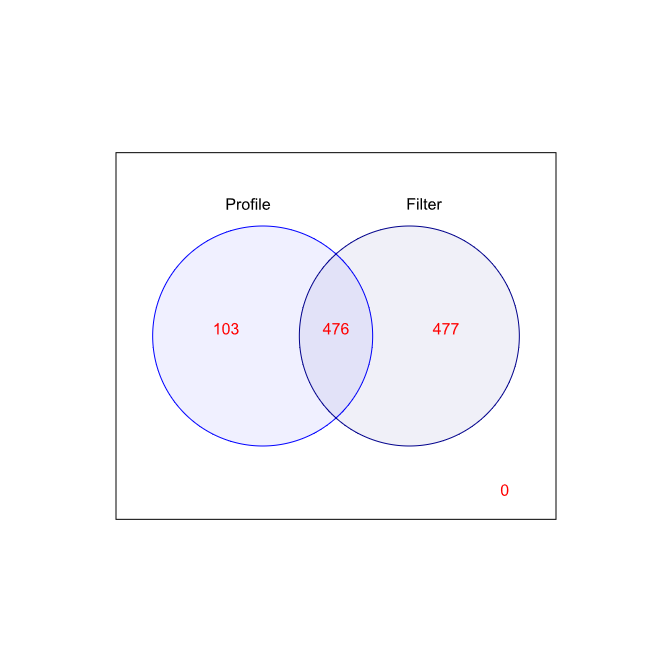**  **(D)**  **82.2%**  **45.0%**  **9.8%**  **45.2%** | 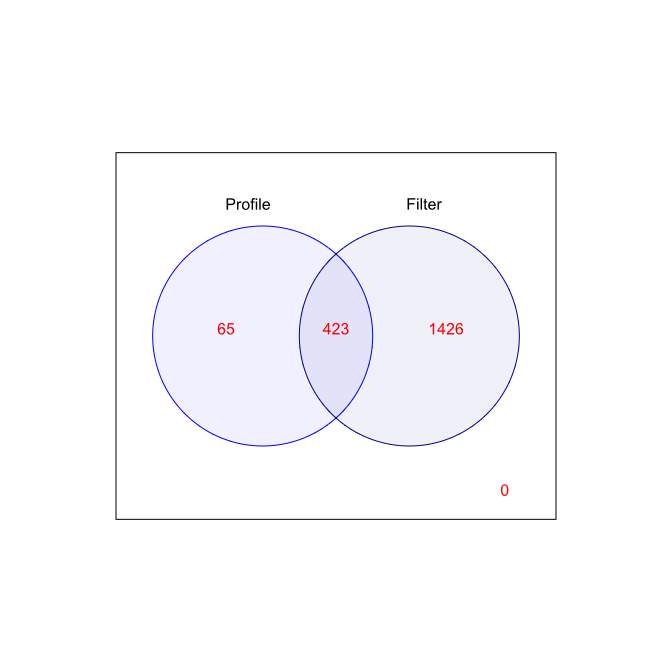  **86.7%**  **22.1%**  **3.4%**  **74.5%** |

**SI-Figure 7:** A) PCoA, B) Alpha-diversity Chao1 (richness) and Shannon (evenness), C) ten most abundant bacterial phyla, resp. fungal classes, D) Venn diagram of common and unique bacterial and fungal genera, green numbers indicate percentage of genera out of the total number of snow genera

The bacterial and fungal communities from air filters and the snow profile differ significantly (ANOSIM bacteria p=0.001, R=0.325, fungi p=0.001, R=0.287). The PCoA plot shows a grouping of samples into air and snow, with some bacterial air and snow samples mixing. Within the complete bacterial dataset, the 140cm and 300cm snow layer were most similar to air. The air filters 24F, 29F, 30F, 31F and 32F cluster most closely to a cluster of most snow samples. For the fungal dataset, the 10cm snow sample was outstanding within the air dataset, while the filters F16 and F24 were closest to the snow dataset.

Alpha diversity differs significantly between air filter and snow (wilcoxon bacteria chao1 p>0.0001, Shannon p=0.0002, fungi chao1 p<0.0001, Shannon p <0.0001) with higher median and maximum values in the air filter samples.

The bacterial community featured 45% of genera unique to the air filters and 45% common in the profile and the air filters, but only 9.8% unique to the snow profile, resp. 82.2% of the snow genera were also present in air. In the fungal community 74.5% of the genera were unique to the air filters while only 3.4% were unique to the snow profile, and 22.1% of genera occurred in air and snow, resp. 86.7% of the snow fungal genera also occurred in the air.

Within the ten most abundant bacterial phyla, the most prominent overall trend was the relative occurrence of more than twice as much Actinobacteria in the air-filters (air 28.09%, snow 10.50%), as second prominent, Proteobacteria were more abundant in the snow profile (snow 39.15%, air 20.54%). Relative abundances of Bacteroidetes (air 10.11%, snow 12.69%), Chloroflexi (air 1.16%, snow 1.24%), Firmicutes (air 30.29%, snow 26.91%), Gemmatimonadetes (air 0.74%, snow 0.66%) and other less abundant phyla were similar in air and snow. Whereas Cyanobacteria appeared to be twice as common in snow (air 2.56%, snow 4.25%), Acidobacteria were more abundant in air (air 2.64%, snow 1.41%).

For the fungi Dothideomycetes (air 19.17%, snow 27.16%), Microbotryomycetes (air 1.35%, snow 7.79%), Lecarnomycetes (air 0.93%, snow 10.45%) and Ustligainomycetes (air 0.32%, snow 9.14%) were more abundant in snow, while Eurotiomycetes (air 27.34%, snow 9.75%) and Sordariomycetes (air 16.49%, snow 7.67%) were more abundant in air. Agaricomycetes (air 20.79%, snow 16.85%) and Leotiomycetes (air 5.97%, snow 4.19%) occured at comparable relative abundances in air and snow.

| **Bacteria** | | **Fungi** |  |
| --- | --- | --- | --- |
| 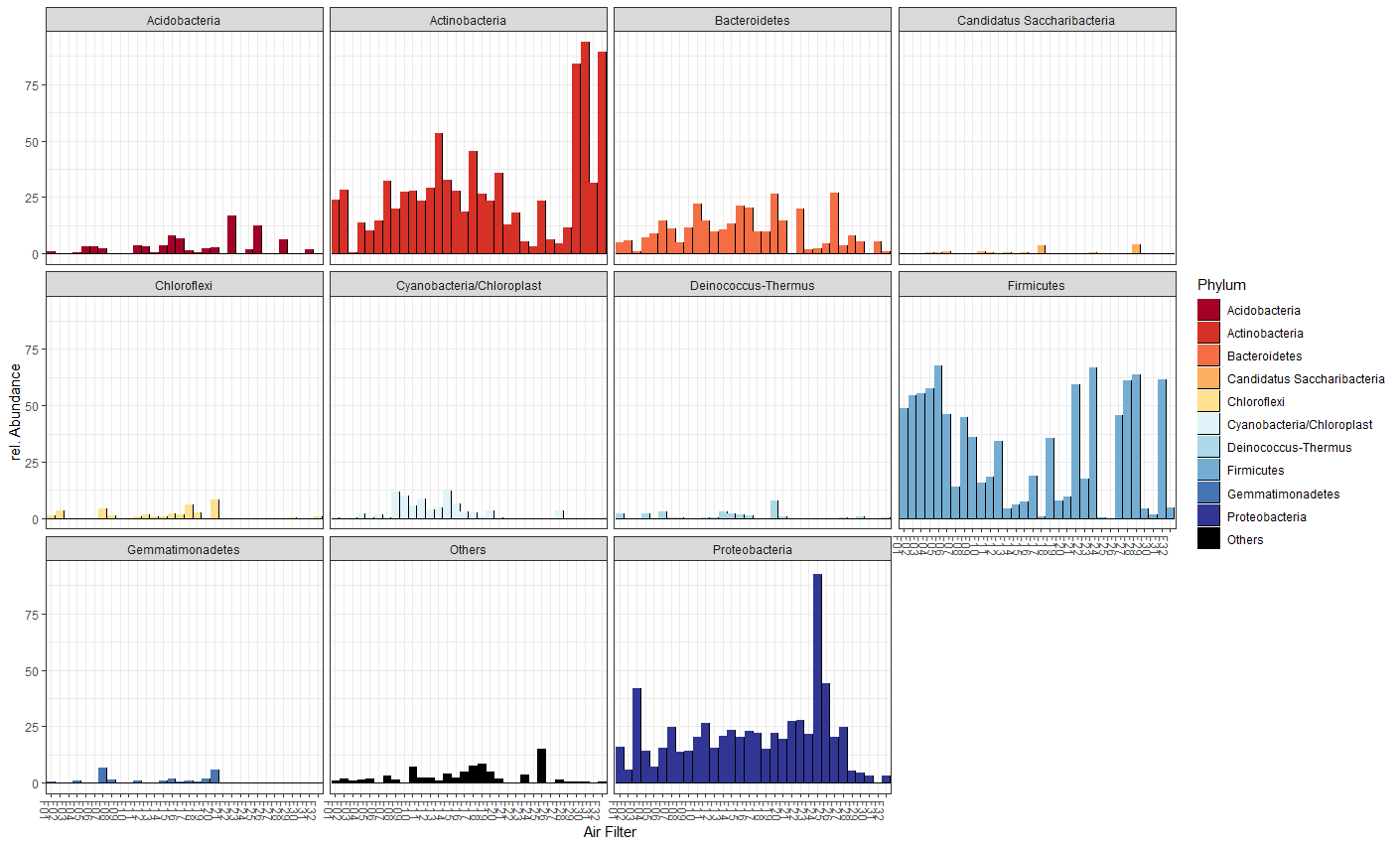  **(A)** | | 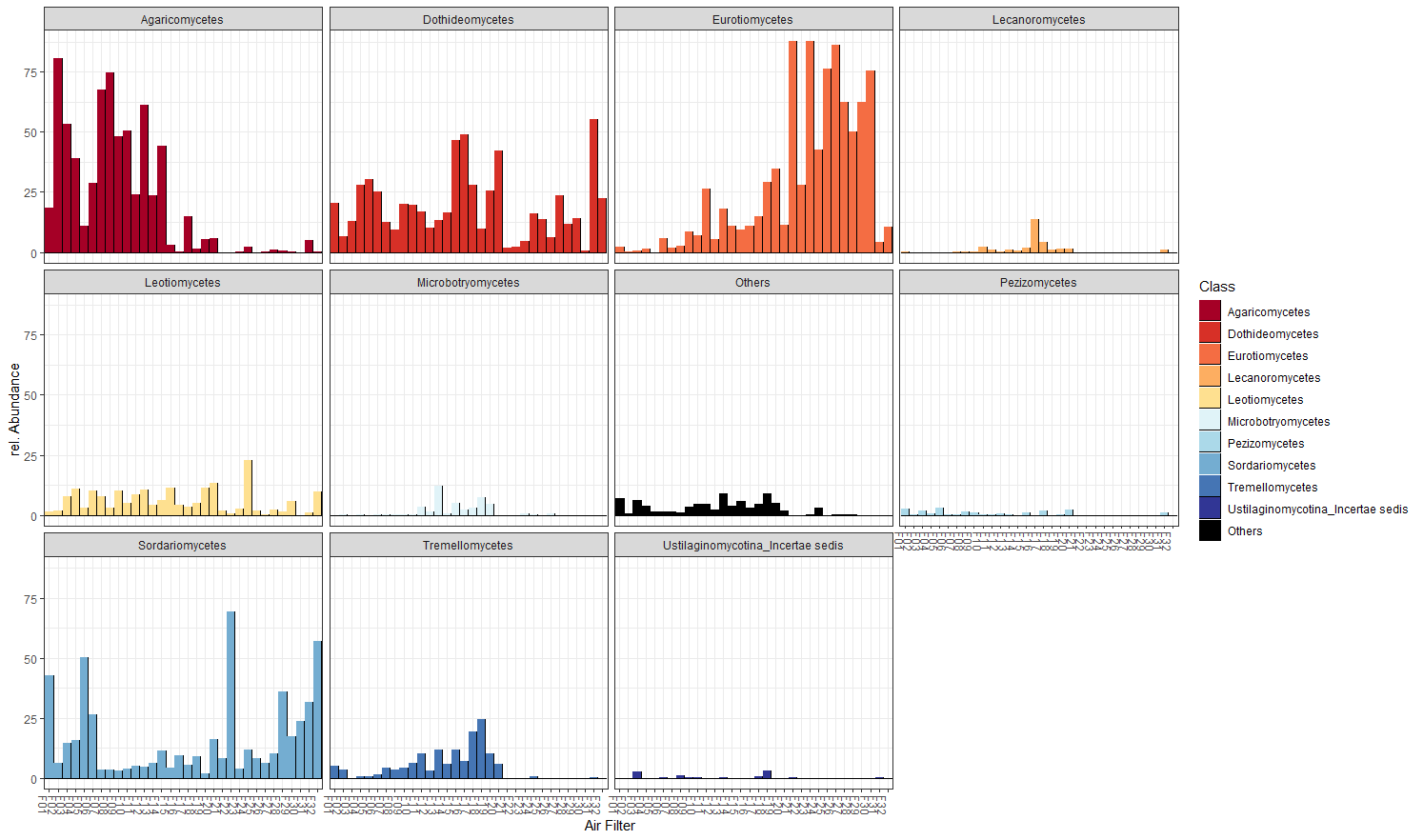 |  |
| 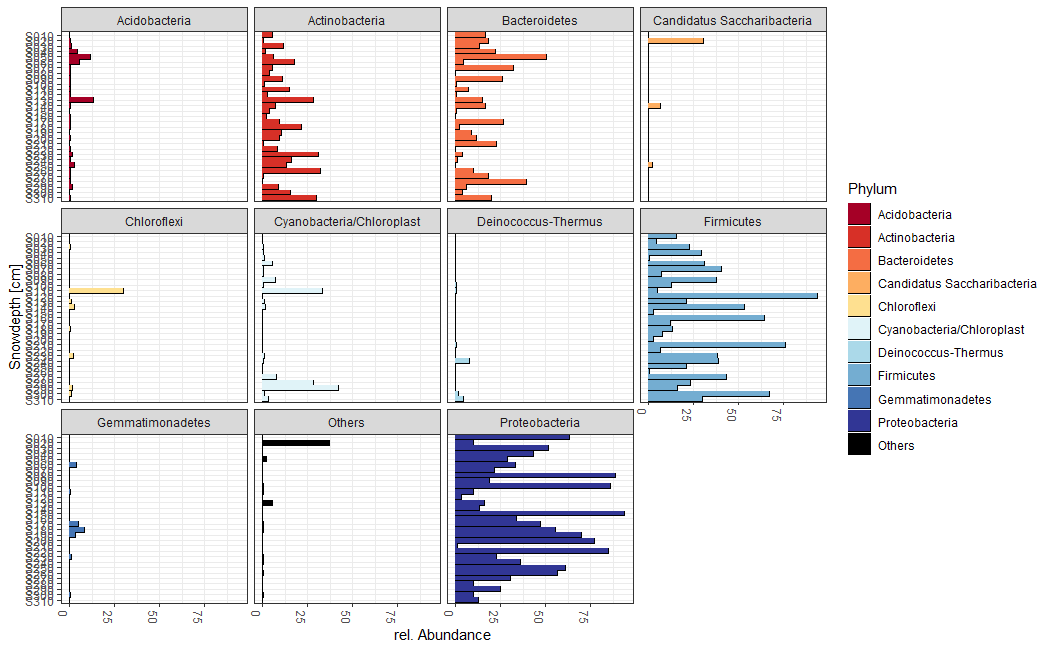  **(B)** | | 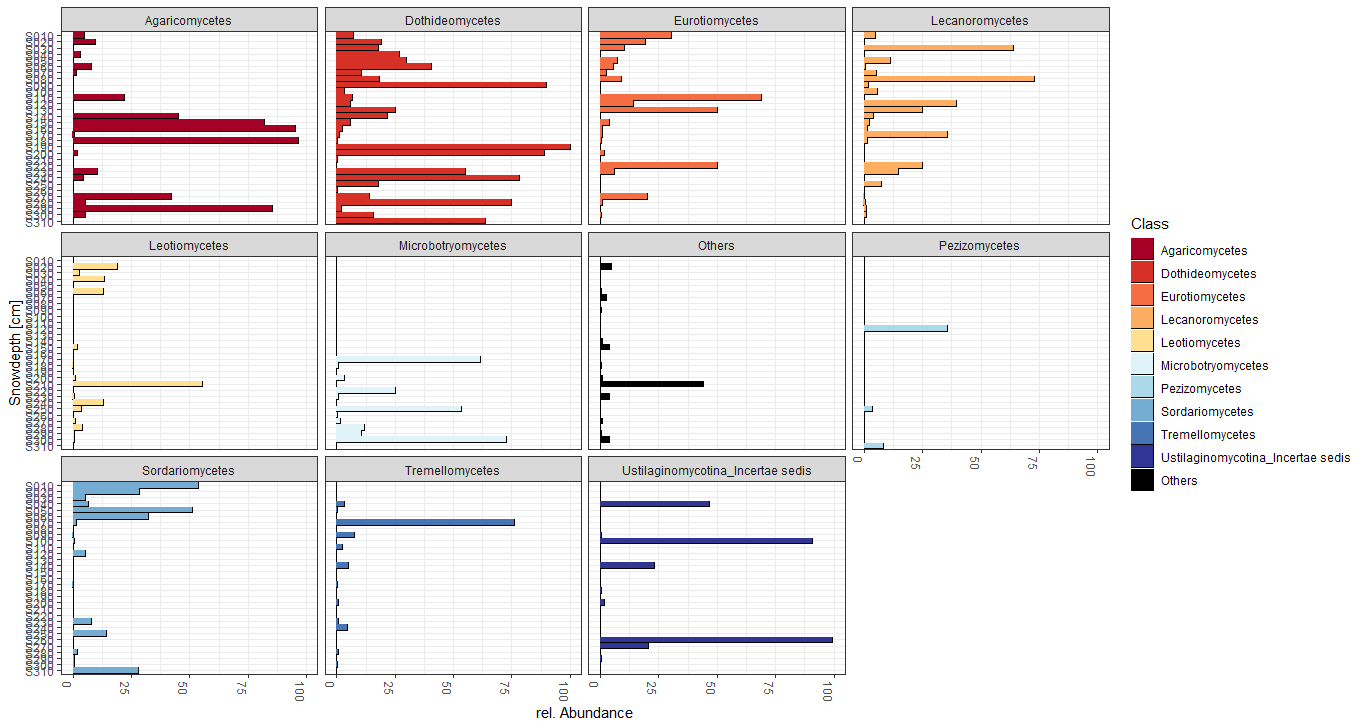 |  |
| 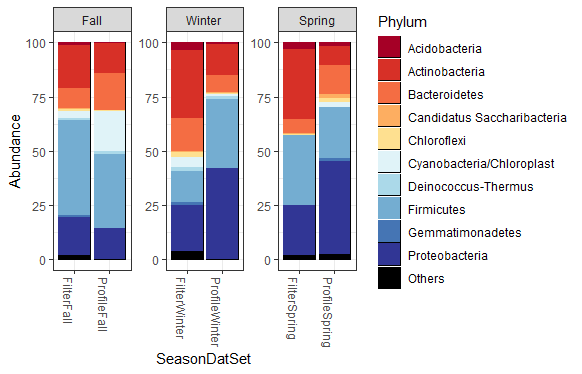  **(D)**  **(C)** | 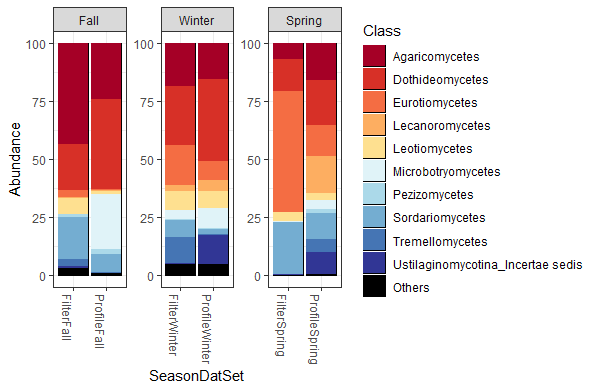 | | |
| \| 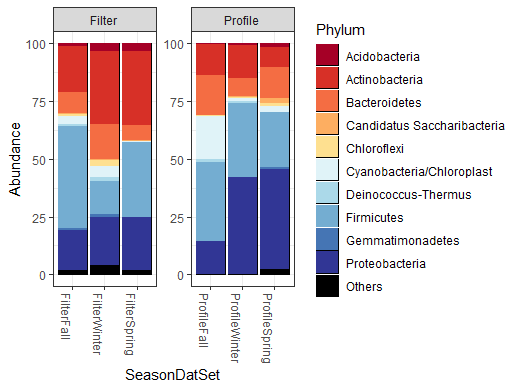 \| 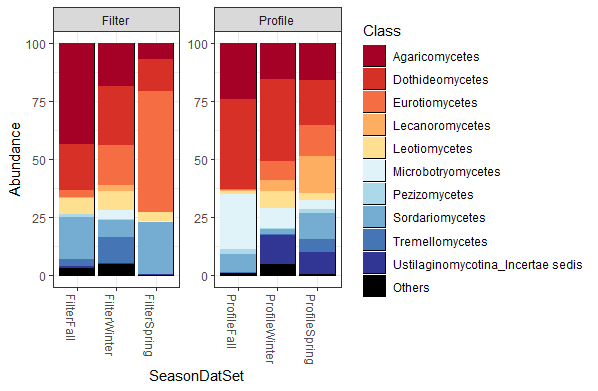 \| \| --- \| --- \| |  | | |

**SI Figure 8:** Temporal succession of A) bacterial phyla, B) fungal classes, C) bacterial phyla and fungal classes by season and sample type , D) bacterial phyla and fungal classes by sample type and season
